# Wnt/β-Catenin Pathway-Regulated Fibromodulin Expression Is Crucial for Breast Cancer Metastasis and Inhibited by Aspirin

**DOI:** 10.3389/fphar.2019.01308

**Published:** 2019-11-25

**Authors:** Fahim Ullah Khan, Nana Yaa Gyaama Owusu-Tieku, Xiaoyong Dai, Kewei Liu, Yanping Wu, Hsiang-I Tsai, Hongbo Chen, Chunhui Sun, Laiqiang Huang

**Affiliations:** ^1^School of Life Sciences, Tsinghua University, Beijing, China; ^2^Shenzhen Key Laboratory of Gene and Antibody Therapy, Center for Biotechnology and Biomedicine, State Key Laboratory of Chemical Oncogenomics, State Key Laboratory of Health Sciences and Technology (prep), Division of Life and Health Sciences, Graduate School at Shenzhen, Tsinghua University, Shenzhen, China; ^3^Precision Medicine and Healthcare Research Center, Tsinghua-Berkeley Shenzhen Institute (TBSI), Shenzhen, China

**Keywords:** fibromodulin (FMOD), gene expression, Wnt/β-catenin pathway, cancer metastasis, aspirin, anti-cancer effects

## Abstract

Emerging evidence suggests that fibromodulin (FMOD), an extracellular matrix protein, is associated with cancer, and yet little is known about the regulation of FMOD expression and its role in cancer metastasis. Aspirin, a classic anti-inflammatory drug, has been indicated to offer anticancer benefits, but its action targets and mechanisms remain obscure. In the present study using cell lines, animal model and database analysis, we show that FMOD is crucial for breast cancer cell migration and invasion (BCCMI) *via* activation of ERK; expression of FMOD is regulated positively by the Wnt/β-catenin pathway, wherein the β-catenin/TCF4/LEF1 complex binds the FMOD promoter to transcribe FMOD. Aspirin inhibits BCCMI by attenuating Wnt/β-catenin signaling and suppressing FMOD expression *via* inhibiting deacetylation of β-catenin by histone deacetylase 6 (HDAC6) leading to β-catenin phosphorylation and cytoplasmic degradation. Moreover, expression of the transcriptional complex components β-catenin/TCF4/LEF1 is upregulated by the Wnt/β-catenin pathway, constituting positive feedback loops that amplify its signal output. Our findings identify a critical role of FMOD in cancer metastasis, reveal a mechanism regulating FMOD transcription and impacting tumor metastasis, uncover action targets and mechanism for the anticancer activity of Aspirin, and expand the understanding of the Wnt/β-catenin pathway and tumor metastasis, which are valuable for development of cancer therapeutics.

## Introduction

Breast cancer (BC) occurs commonly in women and is amongst the deadliest cancers (Center for Disease Control and Prevention (CDC), 2017; Wang et al., 2014b) as aggressive forms of BC are characterized by cells with high proliferation, migration, and invasion potential (Lambert et al., 2017) leading to cancer metastasis, the most common cause of cancer deaths. Therefore, breast cancer especially its invasive progression awaits to be better understood so that new therapeutic targets and agents can be identified for the development of anti-metastatic therapies (Eckhardt et al., 2012). The dynamic extracellular matrix (ECM) is known to regulate diverse cellular functions, including proliferation, migration, differentiation, and play crucial roles in normal physiology and many pathological processes including cancer (Kim et al., 2011; Bonnans et al., 2014). The ECM is recognized as an important regulator and tumor microenvironment in breast cancer, and many ECM components including proteoglycans play key roles in BC progression and metastasis (Insua-Rodriguez and Oskarsson, 2016).

Fibromodulin (FMOD) is a small leucine-rich proteoglycan ECM protein, and is a member of a family of secreted proteoglycans that play important roles in signaling, collagen fibrillogenesis, cell migration, adhesion, growth, differentiation, and apoptosis (Pietraszek-Gremplewicz et al., 2019), while individual members have different functions and effects on cancer (Iozzo and Sanderson, 2011; Edwards, 2012). FMOD is present in a variety of connective tissues such as cartilage, sclera, tendon, skin, and corneas; its function is associated with ECM organization due to the protein’s ability to interact with collagen (Choudhury et al., 2010); and it may be a key component in the microenvironment for various physiol-pathological processes. In a carcinoma model, FMOD controls the stoma matrix structure which affects the internal and external fluid stability of the carcinoma stroma (Oldberg et al., 2007). FMOD was suggested to be a tumor-associated antigen in chronic lymphocytic leukemia (CLL) (Mayr et al., 2005); in clinical samples obtained from prostate cancer patients, significant variations of FMOD expression were observed between benign and malignant tissues (Reyes et al., 2016); and a recent study implicated FMOD in glioma cell migration (Mondal et al., 2017).

The Wnt signaling pathway is a major signaling pathway that normally mediates and regulates an array of cellular processes including proliferation, differentiation, migration, adhesion, and epithelial–mesenchymal transition (EMT) and is aberrantly activated in many human cancers and important for tumor initiation, growth and metastasis (Larue and Bellacosa, 2005; MacDonald et al., 2009; Anastas and Moon, 2013). The Wnt signaling pathway comprises of three pathways: the canonical Wnt pathway (the Wnt/β-catenin pathway), the non-canonical planar cell polarity (PCP) pathway, and the Ca^2+^ signaling pathway (Wang et al., 2014a). In the canonical Wnt/β-catenin pathway, β-catenin (CTNNB1) is ever-present and expressed as a major signal transducer. When the Wnt/β-catenin pathway is inactive, β-catenin forms a complex with E-cadherin, α-catenin, adenomatous polyposis coli (APC) protein and actin filaments (Silva-Garcia et al., 2014). The β-catenin in the complex is then phosphorylated by glycogen synthase kinase 3 α and β (GSK-3α and GSK-3β) and tagged for ubiquitination and degradation. When the Wnt β-catenin pathway is activated by the binding of the Wnt protein to the Frizzled and LRP5/6 Wnt receptors, β-catenin escapes the degradation complex and accumulates in the cytoplasm. β-catenin then translocates to the nucleus, where it binds to transcription factors T cell factor4 (TCF4), and lymphoid enhancer factor1 (LEF1), leading to the up-regulation of target genes, such as cell cycle regulators, Cyclin D1 and c-Myc, whose promoters are directly activated by the β-catenin/TCF4/LEF1 complex. As a result, the accumulation of the cytoplasmic β-catenin has been implicated as a source for oncogenic signaling (Orford et al., 1997; Tetsu and McCormick, 1999).

Aspirin, also known as acetylsalicylic acid (ASA), is one of the best known nonsteroidal anti-inflammatory drugs (NSAIDs) which has long been widely used for the treatment of pain, fever, and inflammatory conditions. The regular use of Aspirin has been shown to reduce the risk of developing colorectal, breast, esophageal, lung, stomach, and ovarian cancers (Cuzick et al., 2009). Aspirin has been shown to have an effect on angiogenesis, inhibit cell growth, induce apoptosis, and limit tumor metastasis. Some epidemiological studies determined that the regular use of Aspirin after a cancer diagnosis improves patient’s prognosis for survival which suggests that Aspirin might be used as an adjunct cancer therapeutic (Langley et al., 2011). Various mechanisms and targets of Aspirin action have been suggested by previous studies in different cancers but are still poorly understood (Dihlmann et al., 2003; Maity et al., 2015; Drew et al., 2016; Bilani et al., 2017; Chen and Holmes, 2017).

How FMOD expression is regulated, what role FMOD plays in cancer metastasis, how Aspirin exerts anticancer effects, and what mechanistic parts of the Wnt/β-catenin signaling pathway are involved remain as important outstanding issues in biology and cancer therapy. In the current study, we have sought to address these questions in the context of breast cancer. Our investigations are focused on molecular and cellular analysis using highly metastatic human and mouse breast cancer cell lines and a non-tumor cell line, and also extended to an *in vivo* mouse model of human breast tumor xenografts and analysis of clinical data from databases, in conjunction with various approaches and technical methods.

We find that the expression of FMOD is regulated positively by the Wnt/β-catenin pathway, in which nuclear β-catenin in complex with TCF4/LEF1 mediates transcription of FMOD, while β-catenin phosphorylation and subcellular localization is regulated by HDAC6 acting on β-catenin, wherein HDAC6 deacetylates β-catenin causing its dephosphorylation and nuclear translocation. Meanwhile, we find that FMOD plays an essential role in breast cancer cell migration and invasion (BCCMI) *via* promoting ERK activation, and thus FMOD, as a transcriptional target gene of the Wnt/β-catenin pathway, mediates the promotive effects of the pathway on BCCMI. Furthermore, we find that Aspirin inhibits BCCMI by suppressing FMOD expression through hampering Wnt/β-catenin signaling *via* inhibiting HDAC6 to enhance acetylation of β-catenin, causing its phosphorylation and cytoplasmic degradation. Thus Aspirin modulates the Wnt/β-catenin pathway, with HDAC6 as a direct target protein, and FMOD as a downstream transcriptional target gene in cancer metastasis, which reveals a significant link between regulation of FMOD with Aspirin action. In addition, expression of TCF4, LEF1 and β-catenin is upregulated by the Wnt/β-catenin pathway, constituting positive feedback loops.

## Materials and Methods

### Cell Culture and Reagents

Human breast cancer MDA-MB-231 cells, mouse breast cancer 4T1 cells, and human embryonic kidney HEK 293T cells were obtained from the American type culture collection (ATCC). MDA-MB-231 (Triple negative highly invasive human breast cancer cell line) cells were cultured in Leibovitz L-15 Medium supplemented with 10% FBS, 100 U/ml penicillin, and 100 mg/ml streptomycin without CO_2_ at 37°C. Mice breast cancer cell line 4T1 cells were maintained in RPMI-1640 supplemented with 10% FBS, 100 U/ml penicillin, and 100 mg/ml streptomycin in 5% CO_2_ at 37°C. Human embryonic kidney HEK 293T cells were grown in DMEM supplemented with 10%FBS, 100U/ml penicillin, and 100 mg/ml streptomycin in 5% CO_2_ at 37°C. Transfection of the plasmids was performed with Lipofectamine 2000 transfection reagent (Invitrogen) according to the manufacturer’s protocol.

### Chemicals

Aspirin (Sigma, purity 99%), Lithium Chloride (Sigma, LiCl purity ± 98%), and B.D Matrigel were purchased from Sigma-Aldrich. Dynabeads Protein G magnetic beads for ChIP assay were purchased from *Invitrogen*. HRP conjugated, *fluorescently-labeled s*econdary antibodies and the ECL detection system for western blot were purchased from KPL (Gaithersburg, *MD*, *USA*).

### Plasmids

To generate the expression plasmid for human FMOD, cDNAs were PCR amplified, then cloned into a pflag CMV2 vector. For constitutive expression, FMOD was subcloned into pBoBi lentivirus expression plasmids to produce the lentivirus, then recombinant pBoBi FMOD was cotransfected with pMDLg/pRRE, pRSV-Rev, pVSV-G into HEK 293T cells, and culture supernatants containing the virus were collected 48 h and 72 h after transfection (primers as shown in **Supporting Information Table 3**). Wnt-3a gene cDNA was PCR amplified using prime star mix(TAKARA) and cloned into the pflag-CMV2 vector. The PCR cycles were: 95 °C for 3 min 1 cycle, 95 °C for (denaturing) 30 s, 55 °C for (annealing) 30 s, 72 °C for (extension) 1 min 35 cycles and 72 °C for 5 min and then hold at 4 °C (primers as shown in **Supporting Information Table 3**). DNA fragments (+200–1,000 BP) corresponding to the potential FMOD promoter constructs were amplified from human genomic DNA from MDA-MB-231 cells and were used as a template when performing polymerase chain reaction with FMOD F.P and FMOD R.P (primers as indicated in **Supporting Information Table 3**). The PCR cycles were 95 °C for 5 min 1 cycle, 95 °C for (denaturing) 20 s, 58 °C for (annealing) 20 s, 72 °C for (extension) 1 min 35 cycles and 72 °C for 7 min and then held at 4 °C. The PCR product was digested with XhoI and NcoI restriction enzymes and cloned into PGL 3 Enhancer luciferase reporter vector (Promega). The promoter constructs +200–1,000/1 were cloned as previously described. The promoter construct +200–1,000/1 (ΔTB1), +200–1,000/2 (ΔTB2), +200–1,000/3 (ΔTB 3) and +200–1,000 (ΔTB1/2/3) were constructed with deletion of the TCF4 core consensus binding site from TB1 (nt-292–286), TB2 (nt-556–552), and binding site 3 position TB3 (nt-654–658) or TCF4 binding site 1/2/3 (triple binding sites) deletion respectively.

### Cell Migration and Invasion

Twenty-four transwell chambers were used to monitor BC tumor cell migration and invasion. Briefly, 1.5 µg of Wnt-3a-pflag-cmv2 plasmid was used for endogenous activation of the canonical β-catenin signaling pathway (Ring et al., 2014). To induced the β-catenin signaling pathway, breast cancer cells were treated with 10 mM LiCl for 6 h, then treated with DMSO or 5 mM Aspirin for 24 h and then trypsinized, and transferred to an 8-µM pore size membrane transwell chamber containing media without FBS. The growth medium containing 20% FBS was placed in the lower chamber of the transwell chamber. After 24 h incubation, cells on the upper side of the membrane were removed by wiping with a cotton swab and stained with crystal violet, and photomicrographed. For the invasion assay, 8-µm pore size membrane transwell chambers were coated with Matrigel (BD). The rest of the assays were performed as previously described. For quantification, the number of migrated and invaded cells on the other side of the membrane were extracted with 33% acetic acid. The absorbance of the eluted stain was determined at 570 nm (Fong et al., 2003; Iwai et al., 2010; Wang et al., 2014b).

### Real-Time PCR Analysis

Total RNA were prepared using the RNA ISO Plus kit (TaKaRa), and a total of 1 µg of RNA was reverse-transcribed into cDNA using the Primescript RT Reagent Kit (Takara) according to the manufacturer’s instructions. Constructed cDNA was then subjected to quantitative PCR analysis using Fast Start Universal SYBR Green Master (ROX; Roche). The PCR setting used were as follows: 95 °C for 5 min for Hot-Start DNA Polymerase Activation, 40 cycles at 95 °C for 15 s (denaturing) and 60 °C for 1 min (annealing), and one cycle at 95 °C for 15 s, 60 °C for 1 min, and 95 °C for 15 s. All of the target gene expression levels were normalized using GAPDH, and the data was expressed as student t-test Two-way ANOVA test using means ± SD of 3 to 8 independent samples. The sequences of primers used is listed in **Supporting Information Table 2**.

### Lentiviral shRNA Cloning, Production, and Infection

Constructs expressing shRNA against target proteins FMOD, β-catenin, TCF4, and LEF1 were generated by subcloning the following oligonucleotides into a pSilencer 2.1-U 6 hygro vector, with a BamHI/HindIII restriction site, and then shuttled into the FG12 vector. Primers are listed in **Supporting Information Table 1**. To produce the shRNA lentivirus, the recombinant FG12 vector was cotransfected with pMDLLg/pRRE, pRSV-Rev, and pVSV-G into HEK 293T cells. Subsequently, the culture supernatants which contained the virus were collected 48 h and 72 h after transfection. For infection with the lentivirus, MDA-MB-231 cells were cultured with the lentiviral solution and 1 µg/ml Polybrene (Sigma) for 24 h at a multiplicity of infection (MOI) of 20–25.

### Immunofluorescence Staining

Following lentiviral or Aspirin treatment, MDA-MB-231 or 4T1 cells were fixed onto slides with 4% paraformaldehyde for 30 min and permeabilized with 0.4% Triton X-100 and blocked with 3% BSA for 1 h. The cells were then incubated with primary antibody (dilution 1:200) against β-catenin overnight at 4 °C, following which, FITC- or Cy3-conjugated goat anti-rabbit IgG antibodies were used as the secondary antibody. The cells were incubated with the FITC-Conjugation secondary antibody (dilution 1:50) for 2 h. To visualize the cell’s nucleus, DAPI (dilution 1:5,000 Invitrogen) was used. Sections were observed using an Olympus Laser Scanning Confocal Microscope with imaging software (Olympus Fluoview FV1000, Tokyo, Japan).

### Western Blot Analysis

Cells or tumor tissues were lysed in a buffer containing 20 mM Tris–HCl pH 7.4, 150 mM NaCl, 1 mM EDTA, 1 mM EGTA, 1% Triton X-100, 2.5 mM sodium pyrophosphate, 1 mM β-glycerol phosphate, 1 mM sodium orthovanadate, 2 µg/ml leupeptin, and 1 mM PMSF. The proteins were fractionalized by 10% and 12% polyacrylamide SDS page and electrophoretically transferred to a PVDF (Millipore) membrane. After blocking the membrane with 5% non-fat dry milk for 1 h at room temperature, the membrane was washed 3 times for 7 min intervals with 1XTBST and then incubated with primary antibodies against FMOD (Polyclonal 2621108 Temecula California ABT 124), GSK-3β (27C10) Rabbit Mab, Phospho GSK-3β (Ser 9) (5b3) Rabbit Mab, β-catenin Rabbit primary antibody (D10A8), Phospho β-catenin (Ser 33/37/Thr 41) antibody, anti-ERK 1/2, phospho-ERK1/2 (Thr202/Tyr204) (9102S, 4370S CST); TCF4 (CST#2569), LEF1 (CST), Acetyl-β-catenin (Lys 49) (CST), GAPDH (Santa Cruz), Lamin B (Santa Cruz), and anti-Ubiquitin (Santa Cruz) overnight at 4 °C. After which, the blots were incubated with horseradish peroxidase (HRP) conjugated secondary antibodies (Pierce) at room temperature for 1 h. Detection was performed using a Chemiluminescent Western Blot detection kit (cell signaling) (Liu et al., 2013). The protein bands were scanned and the density of the bands were quantified as a ratio to the loading control by using Image J software (Java 8).

### Nuclear and Cytoplasmic Protein Extraction

For nuclear and cytoplasmic protein extraction, treated cells were initially washed and scraped with cold PBS pH 7.4 in 1 ml per 100-mm dishes and subsequently transferred into 1.5 ml Eppendorf tubes. Cell were centrifuged at 3,000 rpm for 3 min at 4 °C, suspended and incubated in 400 ul of buffer A (10 mM HEPES, pH 7.9, 10 mM KCl, 0.1 mM EDTA, 1 mM DTT and protease inhibitor PMSF) on ice for 10 min, and then added to 10 ul of 10% NP-40 to lyse the cells. The supernatant was collected by centrifugation (6,000 rpm at 4 °C for 5 min) to obtain the cytoplasmic fraction. The pellet was resuspended in 50 ul buffer B (20 mM HEPES, pH 7.9, 0.4 M NaCl, 1 mM EDTA, 1 mM DTT and protease inhibitors PMSF) and incubated on ice for 15 min to dissolve the nuclear proteins. The isolate was identified by western blot using Lamin B and GAPDH as controls.

### Luciferase Assay

MDA-MB-231 and HEK 293T cells were seeded onto 12-well plates. When cells grew to 60–70% confluence, the cells were transfected with luciferase reporter plasmid PGL 3 which contained either the wild type or mutant FMOD promoter sequence (sequence > gi|568815597:c203340720-203339621 *Homo sapiens* chromosome 1, GRCh38.p7 Primary Assembly) which was obtained from NCBI as previously described. All the transfections were performed using Lipofectamine 2000 (Invitrogen) according to the manufacturer’s instructions. Cell lysates were used for luciferase assay using a luciferase assay kit (Promega, *Madison, WI, USA*).

### Chromatin Immunoprecipitation (ChIP) Assay

To analyze the binding of β-catenin or TCF4 to the FMOD promoter region, MDA-MB-231 cells were cultured in 100-mm cell culture plates under standard conditions. Cells were fixed with formaldehyde for 15 min and crosslink reactions were then stopped by adding 5 mM glycine for 10 min,following the previously published protocol (Nelson et al.,2006). After washing with PBS, the cells were collected and resuspended in ChIP lysis buffer (50 mM Tris–HCl pH 7.5, 150 mM NaCl, 5 mM EDTA, 0.5% NP-40, 1.0% Triton-X-100, and protease inhibitor cocktail), then sonicated into 200 to 1,000 bp fragments using a previously described protocol (Nelson et al., 2006). Sonicated eluted chromatin materials was then immunoprecipitated using β-catenin Rabbit primary antibody (D10A8), TCF4 (CST#2569) primary antibody Rabbit and IgG Rabbit (Sanat Cruz). PCR was performed on the eluted DNA, and the PCR products were analyzed by 2.3% agarose gel. Primers as shown in **Supporting Information Table 3**.

### Human Breast Cancer Xenograph Study

Six to eight week old female BALB/c nude mice (nu/nu) were obtained from Guangdong Province Medical Animal Centre and maintained and monitored in a pathogenic free environment. The animal study was reviewed and approved by the Administrative Committee on Animal Research of the Graduate School at Shenzhen, Tsinghua University. 6 × 10^6^ MDA-MB-231 cells were suspended in 100 µL L-15 media and then injected into the right flank of each BALB/c nude mouse. After inoculation, the mice were randomly divided into two groups. The experimental group received 75 mg/kg of Aspirin dissolved in PBS (neutralized with equimolar NaOH) which was injected into the mice every day for 7 days while the control group mice received PBS solution. At the time of animal sacrifice, tumors were excised and cells were lysed for western blot, Quantitative Real-time PCR, and immunofluorescence.

### Fluorescent Immunohistochemistry of Tumor Tissue

Formalin-fixed, paraffin-embedded frozen sections (4–10 µm) of tissue obtained from breast carcinoma patients were selected for fluorescent immunohistochemical analysis. The tumor tissue was surgically excised and cut into small sections for further fixation with 4% paraformaldehyde for 2 h. The fixed tumor tissue samples were dehydrated in a series of 10%, 20%, and 30% sucrose in PBS solutions and the samples were then wrapped in Tissue-Tek O.C.T. compound (Sakura Finetek, USA) and frozen at −80 °C for 2 h. The frozen samples were then sectioned into 4–10 µm thick sections. After blocking with 3% BSA/0.2% Triton X-100 in PBS for 1 h, the sections were incubated with anti β-catenin (D10A8), and anti-FMOD antibodies (Polyclonal 2621108 Temecula California ABT124) overnight at 4 °C. FITC conjugated goat anti-rabbit IgG antibody (1:50 Pierce) was used as a secondary antibody. To observe the nucleus, DAPI was used. Sections were observed using an Olympus confocal laser scanning microscope with imaging software (Olympus Fluoveiw FV-1000, Tokyo Japan) (Cao et al., 2015).

### Statistical Analysis

All experiments were repeated a minimum of 3 times and maximum of 8 times and the data are presented as means ± SD. The difference between data groups was evaluated for statistical significance using the Student *t-test* of unpaired data or two-way ANOVA (Prism 4.00; Graph Pad). P values less than 0.05 indicates statistical significance.

## Results

### Aspirin Inhibits Breast Cancer Cell Migration and Invasion Promoted by the Wnt/β-Catenin Signaling Pathway

**Figure 1:**
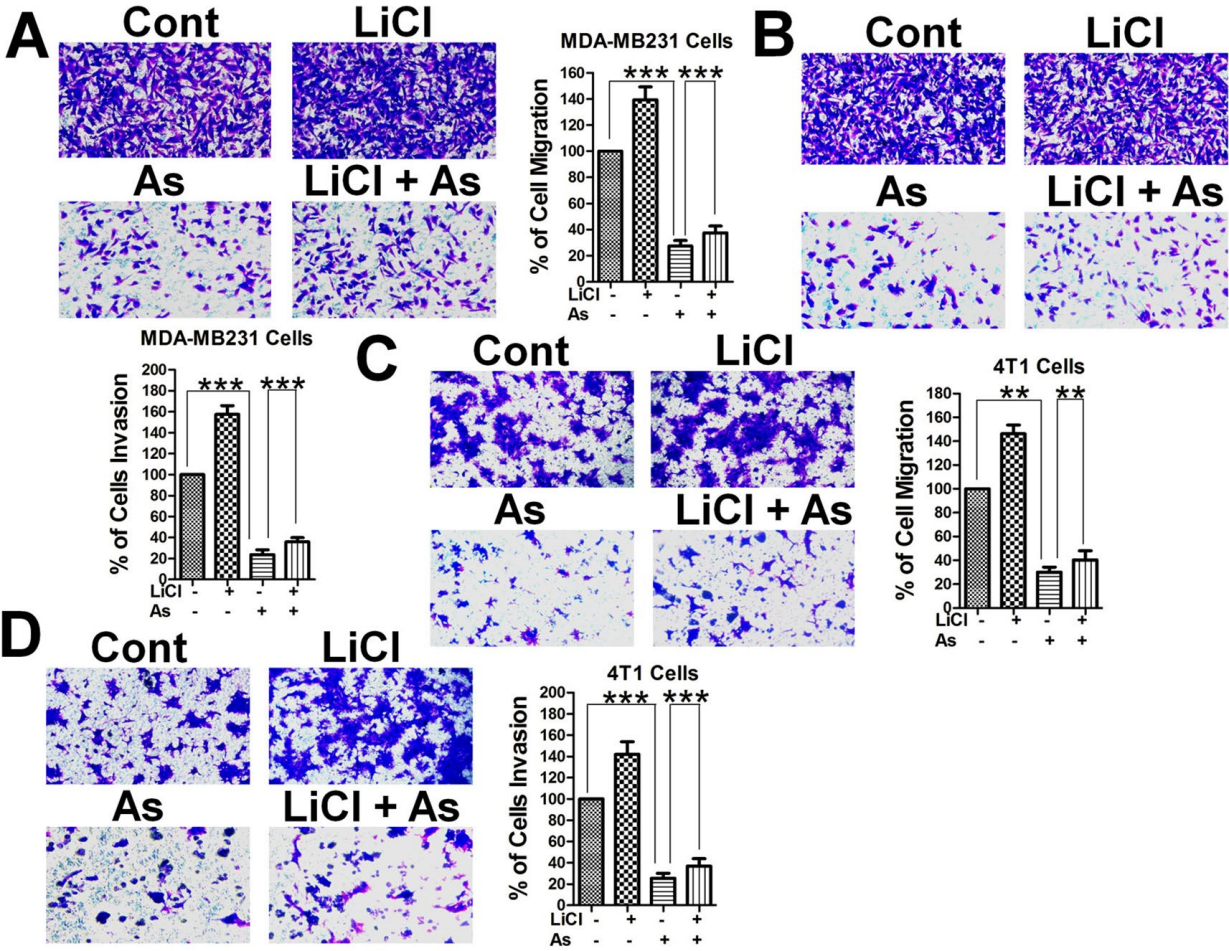
Aspirin inhibits breast cancer cell migration and invasion (BCCMI) promoted by the canonical Wnt/β-catenin pathway. Cells were treated with or without 5 mM Aspirin (AS) or 10 mM LiCl (an activator of Wnt/β-catenin pathway) for 24 h and subjected to migration assay using a transwell chamber or invasion assay using a transwell chamber coated with matrigel. The cells were then fixed and stained with crystal violet, and migrated cells were photomicrographed and quantified. **(A)** Migration and **(B)** Invasion assay with human breast cancer MDA-MB-231 cells. **(C)** Migration and **(D)** Invasion assay with mouse mammary tumor 4T1 cells. Data is shown as the mean ± SD of three independent experiments. Student’s t-test was used for statistical analysis (**P 0.01, and ***P 0.001).

Breast cancer cell migration was suggested to be inhibited by Aspirin (Maity et al., 2015), and to confirm that hypothesis and to explore the possible involvement of the Wnt/β-catenin pathway, we performed transwell migration and invasion assays with highly metastatic MDA-MB-231 human mammary tumor cells and 4T1 mouse mammary tumor cells. Both human (**Figures 1A, B**) and mouse (**Figures 1C, D**) mammary tumor cells treated with Aspirin at 5 mM showed a marked decrease in migration and invasion in comparison to untreated cells. Interestingly, when treated with 10 mM lithium chloride (LiCl), an inhibitor of GSK-3β and activator of the Wnt/β-catenin signaling pathway (Dihlmann et al., 2003; Jansson et al., 2005), both human (**Figures 1A, B**) and mouse (**Figures 1C, D**) mammary tumor cells displayed a marked increase in migration and invasion ability; and in the treatments combining LiCl and Aspirin, the inhibitory effect of Aspirin largely remained while overriding the enhancement by LiCl. These results indicate that the Wnt/β-catenin signaling pathway plays a role in promoting the metastasis of breast cancer, and Aspirin has strong anti-metastatic effects and inhibits breast cancer cell migration and invasion (BCCMI) by acting on the Wnt/β-catenin signaling pathway, likely at some point downstream of LiCl activation.

### FMOD Promotes BCCMI, and FMOD Expression Is Promoted by the Wnt/β-Catenin Pathway and Inhibited by Aspirin

**Figure 2:**
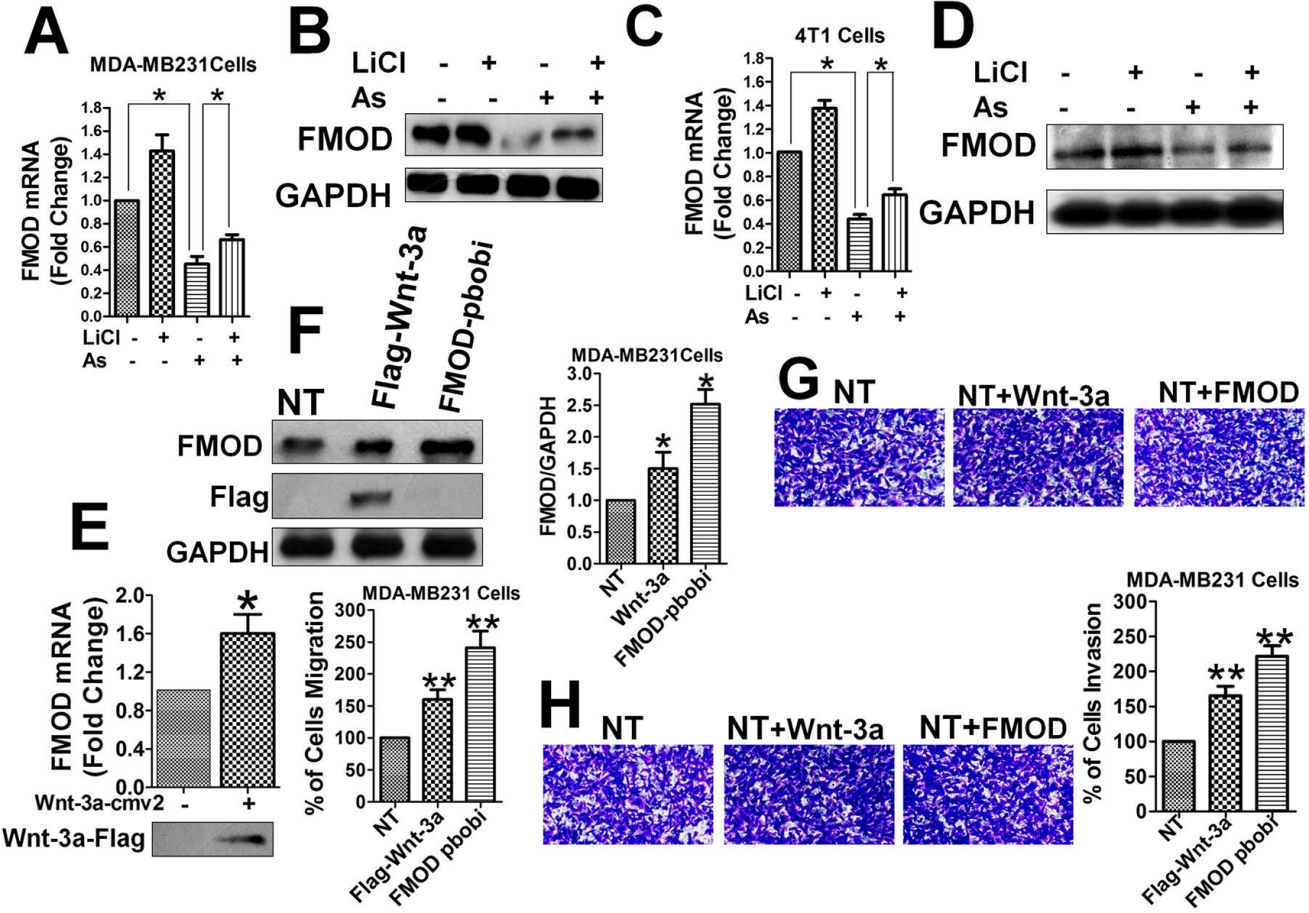
FMOD expression is promoted by Wnt/β-catenin pathway and inhibited by Aspirin, and FMODpromotes BCCMI. MDA-MB-231 cells **(A**, **B**, **E**, **F**, **G**, and **H)** and 4T1 cells **(C**, **D)** were treated withLiCl for 6 h prior to DMSO or 5 mM Aspirin treatment for 24 h. FMOD expression was determined using qPCR (for mRNA) and Western blot (for protein) with antibodies against the indicated protein (see *Materials and Methods*). Cell migration and invasion were assayed as described in **Figure 1**. **(A)** FMOD mRNA level was significantly inhibited by Aspirin. **(B)** FMOD protein level was significantly inhibited by Aspirin. **(C)** FMOD mRNA level was significantly inhibited by Aspirin in 4T1 cells. **(D)** FMOD protein level was significantly inhibited by Aspirin in 4T1 cells. **(E)** Wnt/β-catenin pathway activator Wnt-3a significantly enhanced the FMOD mRNA expression. **(F)** FMOD protein level in MDA-MB-231 cells 24 h after transfection with Wnt-3a-Flag plasmid or FMOD pBOBI lentiviruse increased significantly. **(G)** and **(H)** MDA-MB-231 cells treated with Wnt-3a-Flag and FMOD pbobi showed an increase in migration **(G)** and invasion **(H)**. Data is shown as the mean ± SD of three to eight independent experiments. Student two-tailed t-test was used for statistical analysis (*P 0.05, and **P 0.01).

To understand the role of FMOD gene expression in breast cancer metastasis in relation to Aspirin and the Wnt/β-catenin signaling pathway, we measured the mRNA and protein levels of FMOD, in the presence and absence of interference of the Wnt/β-catenin pathway, by qPCR and Western blot. FMOD mRNA and protein levels were markedly reduced in MDA-MB-231 (**Figures 2A, B**) and 4T1 cells (**Figures 2C, D**) treated with Aspirin. FMOD mRNA and protein levels were significantly upregulated by overexpressing Wnt-3a, an activator of the Wnt/β-catenin signaling pathway (Anastas and Moon, 2013), or by over expressing FMOD itself using a lentivirus construct (FMOD pbobi) in MDA-MB-231 cells (**Figures 2E, F**). These results indicate that expression of FMOD is promoted by the Wnt/β-catenin pathway and suppressed by Aspirin, suggesting a possible role of FMOD in BCCMI.

Next, the direct relationship between the FMOD protein and BCCMI was investigated. Activation of the Wnt/β-catenin signaling pathway by overexpressing Wnt-3a or FMOD in MDA-MB-231 cells enhanced breast cancer cell migration (**Figure 2G**) and invasion (**Figure 2H**). Likewise, LiCl enhanced breast cancer cell motility; but the enhancement could be eliminated by knocking down FMOD, or individual components of the β-cat/TCF4/LEF1 transcriptional complex, or by inhibiting HDAC6 with Trichostatin A (TSA) (**Figures S1A, B**). The inhibitory effects of Aspirin on BCCMI were largely reversed by overexpression of FMOD or HDAC6, but not by Wnt-3a overexpression or LiCl (**Figures S1C, D**). These results suggest that FMOD promotes breast cancer cell metastasis through (being a downstream effector of) the Wnt/β-catenin pathway which is sensitive to Aspirin inhibition, and wherein the gene products play vital, specific and interrelated roles. The results also suggest that the point of action of Aspirin on the Wnt/β-catenin pathway is probably downstream of Wnt and LiCl/GSK-3β but upstream of β-catenin and HDAC6.

### FMOD Is Required for BCCMI and ERK Activation Which Is Mediated by the Wnt/ β -Catenin Pathway and Attenuated by Aspirin

**Figure 3:**
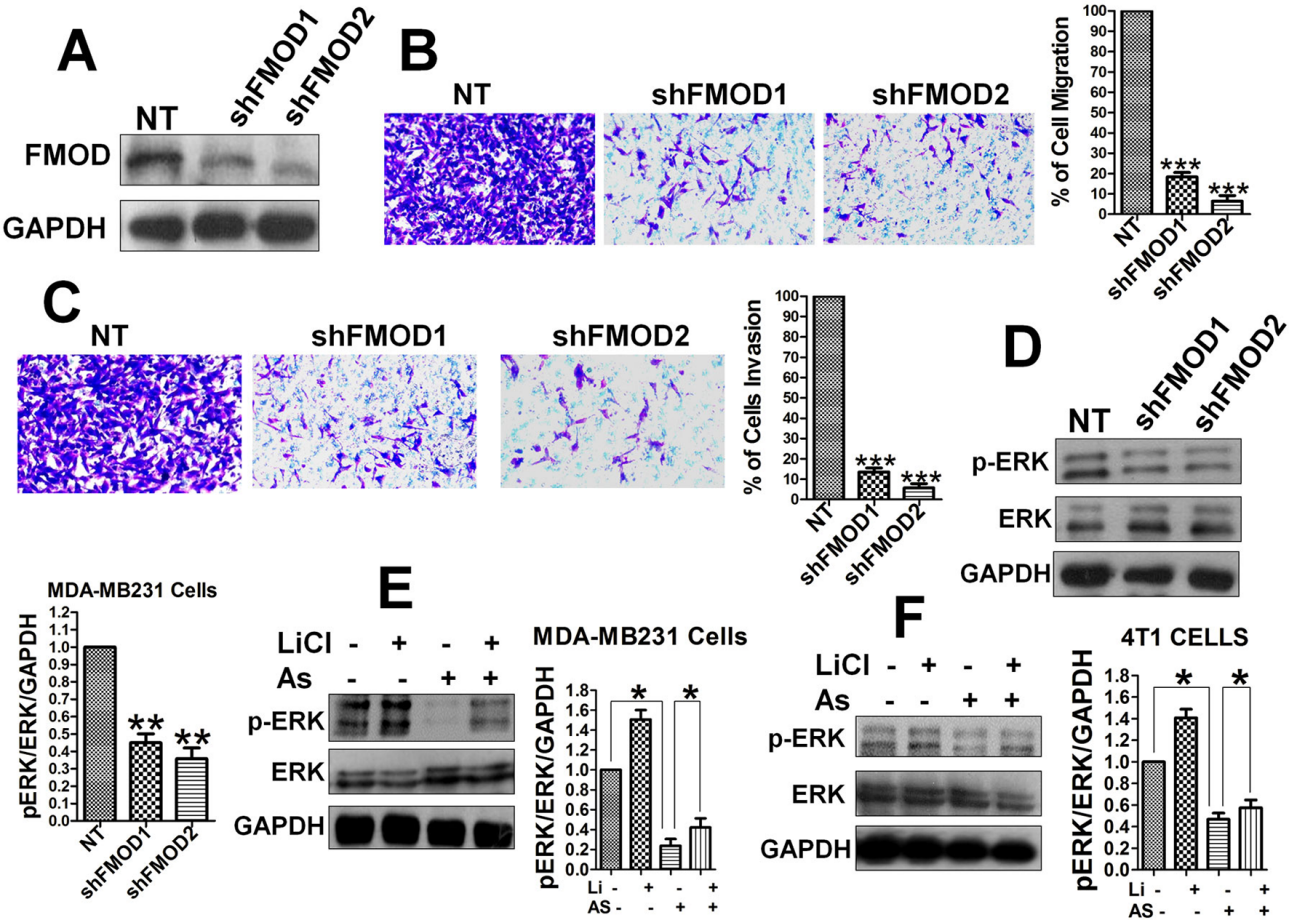
FMOD is essential for BCCMI and ERK activation which are augmented by the Wnt/β-catenin pathway and attenuated by Aspirin. MDA-MB-231 cells **(A**–**E)** and 4T1 cells **(F)** were used. Cell migration and invasion assays were performed as in **Figure 1**, and LiCl and Aspirin treatments were administered as in **Figure 2**. For knockdown with lentivirus-mediated shRNA, cells were grown in L-15 media supplemented with 10% FBS and without antibiotics, then infected with the shRNA-carrying lentivirus for 24 hours in the presence of polybrene (also see *Materials and Methods*). **(A)** Lentivirus-mediated shRNA against FMOD markedly knockdown FMOD protein expression. **(B)** Knockdown of FMOD inhibited migration of the cells. **(C)** Knockdown of FMOD inhibited invasion of the cells. **(D)** Knockdown of FMOD significantly inhibited ERK phosphorylation in the cells. **(E)** Aspirin significantly reduced the ERK phosphorylation in human breast cancer MDA-MB-231cells. **(F)** Aspirin significantly inhibited ERK activation in mouse breast cancer 4T1 cells. Data is shown as the mean ± SD of three independent experiments. Student’s t-test was used for statistical analysis (*P < 0.05, **P < 0.01, and ***P < 0.001).

To determine a direct relationship between FMOD, BCCMI, and ERK activity in MDA-MB-231 and 4T1 cancer cells, FMOD knockdown experiments using lentivirus shRNA were performed. Knockdown of FMOD (**Figure 3A**) led to a significant decrease in the migration (**Figure 3B**) and invasion (**Figure 3C**) potential of the MDA-MB-231 cells, which indicates that FMOD is essential for BCCMI. Together with our preceding results, this suggests that FMOD is associated with the Wnt/β-catenin pathway in regulating BCCMI in which where FMOD is required for promoting BCCMI.

**Figure 4:**
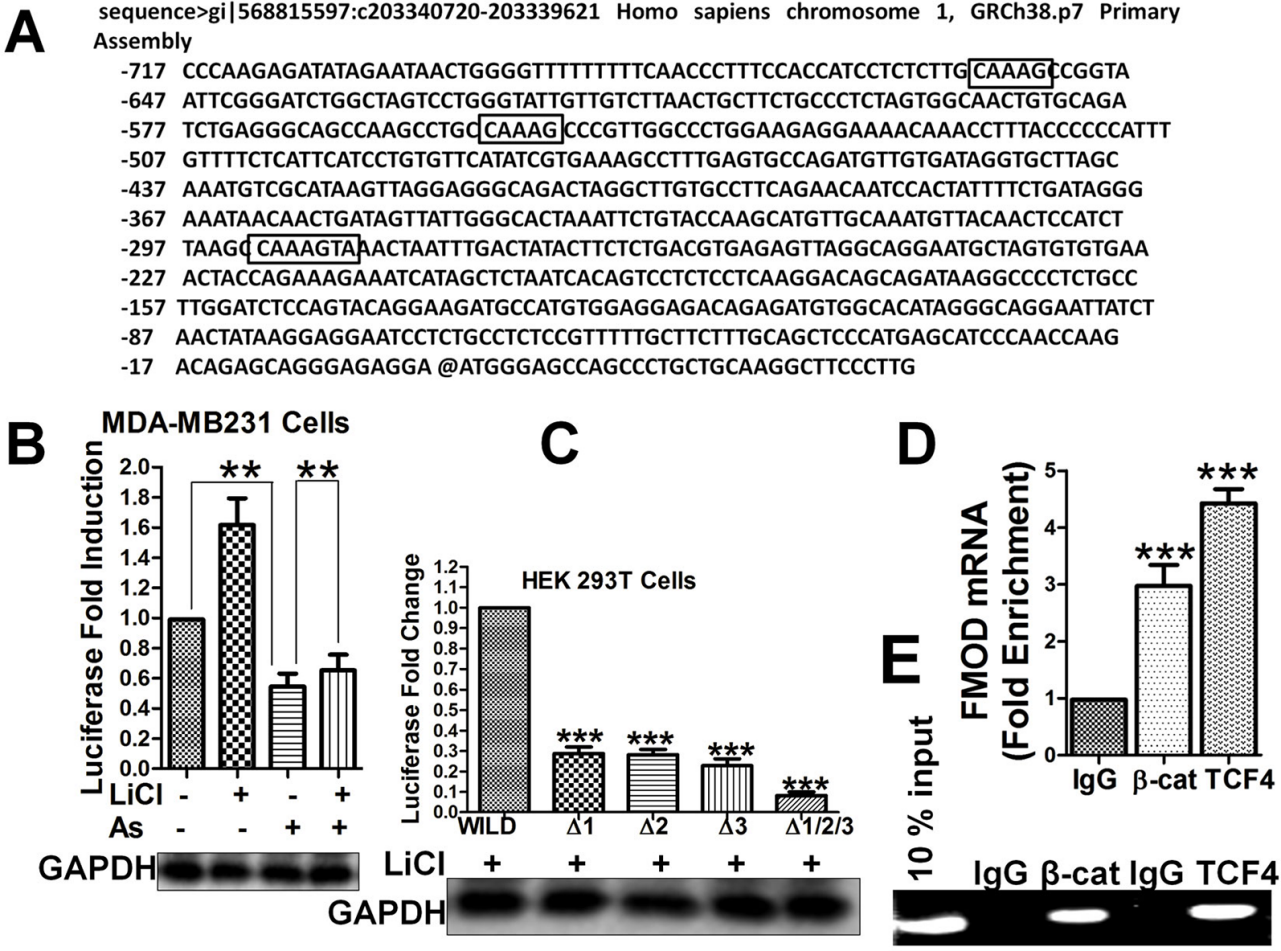
FMOD promoter contains specific sites for binding by β-catenin/TCF4 to activate FMOD transcription which is promoted by the Wnt/β-catenin pathway and inhibited by Aspirin. **(A)** Nucleotide sequence of FMOD promoter sequence gi|568815597:c203340720-203339621 Homo sapiens chromosome 1, GRCh38.p7 Primary Assembly. Three putative β-catenin/TCF4 binding sites are indicated (boxed). **(B)** Quantification of FMOD promoter activity. MDA-MB-231 cells were cotransfected with FMOD-LUC reporter plasmid for 12 hours, and then were treated with or without 10 mM LiCl for 6 hours before treatment with DMSO or 5 mM Aspirin for 24 h. Luciferase activity was measured (see *Materials and Methods*) and normalized to GAPDH. **(C)** Quantification of FMOD promoter activity in HEK 293T cells that had TCF4 binding site core sequence deleted individually or in combination. HEK 293T cells were treated with LiCl in combination, with wild-type or mutant FMOD-PGL3 plasmids. After 24 hours the luciferase activity was measured and normalized to GAPDH. **(D** and **E)** CHIP assay (see *Materials and Methods*) was performed to verify the physical association of endogenous β-catenin and TCF4 with FMOD promoter sequence in MDA-MB-231 cells. ChIPs from fragmented chromatins of MDA-MB-231 cells were incubated with IgG, β-catenin and TCF4 primary antibodies. The purified DNA was analyzed by qPCR using the indicated primers (see SI). Data is shown as the mean ± SD of three to eight independent experiments. Student two-tailed t-test was used for statistical analysis (**P < 0.01, and ***P < 0.001).

As the phosphorylation of ERK1/2 is known to play a role in accelerating BCCMI (Wang et al., 2014b), we measured the level of phospho-ERK1/2 (p-ERK1/2) in MDA-MB-231 cells with FMOD knocked down to determine if ERK1/2 is a downstream effector of FMOD and the Wnt/β-catenin pathway. The level of p-ERK1/2 was markedly reduced in shFMOD2-treated MDA-MB-231 cells (**Figure 3D**); and decreased by Aspirin treatment while increased by LiCl treatment in MDA-MB-231 (**Figure 3E**) and 4T1 (**Figure 3F**) cells; which indicate that ERK activation is dependent on FMOD and the Wnt/β-catenin pathway. The effect of knocking down FMOD expression and the effect of Aspirin on ERK/p-ERK 1/2 are correlated with each other. These results suggest that FMOD-mediated phosphorylation of ERK is inhibited by Aspirin and modulated *via* the β-catenin pathway resulting in the modulation of BCCMI. Our results indicate that FMOD mediates ERK activation to facilitate BCCMI, which is promoted by the Wnt/β-catenin pathway and inhibited by Aspirin.

### β-Catenin and TCF4 Bind FMOD Promoter at Specific Sites for FMOD Transcription Which Is Promoted by Wnt/β-Catenin Pathway and Inhibited by Aspirin

As the Wnt/β-catenin signaling pathway and Aspirin appear to control FMOD expression at the transcriptional level, we tried to identify potential binding sites in the FMOD gene for TCF4, the transcription factor of the Wnt/β-catenin pathway. We analyzed the FMOD promoter region (bp +200 to −1000) for regulatory DNA binding elements and three binding sites that show a high degree of homology to the core consensus sequence of TCF4 binding (5^/^-[A/T][[A/T]CAAAG-3^/^) (Zhang et al., 2011; Henderson et al., 2012) were identified (**Figure 4A**): site 1, nt -292 to -286 (100% homology), site 2, nt -556 to -552 (71.42% homology), site 3, nt -658 to -654 (71.42% homology). Promoter-Luciferase reporter, binding site deletion and ChIP experiments showed that all 3 putative binding sites are important and can bind to the TCF4 protein. Reporter gene expression showed that treatment with Aspirin which inactivates the Wnt/β-catenin pathway decreased the FMOD gene promoter activity and in contrast, treatment with LiCl enhanced FMOD promoter activity (**Figure 4B**). These results suggest that FMOD expression is positively regulated by the Wnt/β-catenin signaling pathway and targeted by Aspirin for inhibition through a transcriptional mechanism. To further test the binding and direct control of FMOD transcription by β-catenin and TCF4, HEK 293T cells with either single (ΔTB1, ΔTB2, and ΔTB3) or triple (ΔTB1/2/3) β-catenin/TCF4 binding site deletion in the FMOD promoter were subjected to LiCl treatment which activates the Wnt/β-catenin pathway to examine the effects of deleting the binding sites on FMOD transcription. Deletion of only one binding site or all three binding sites in the promoter sequence resulted in a dramatic decrease in the transcriptional activity of the FMOD promoter (**Figure 4C**), while the wild-type FMOD promoter was significantly activated by LiCl (**Figures 4B, C**). These results suggest that the TCF4 binding sites are required for β-catenin/TCF4 binding and activity of the FMOD promoter. To confirm the physical association of endogenous β-catenin and TCF4 with the FMOD promoter sequence in MDA-MB-231 cells, ChIP assays were performed and it was determined that the FMOD promoter is occupied by β-catenin and TCF4 in MBA-MB 231 cells (**Figures 4D, E**).

### β-Catenin, TCF4, and LEF1 Are Essential for FMOD Expression, ERK Activity, and BCCMI

**Figure 5:**
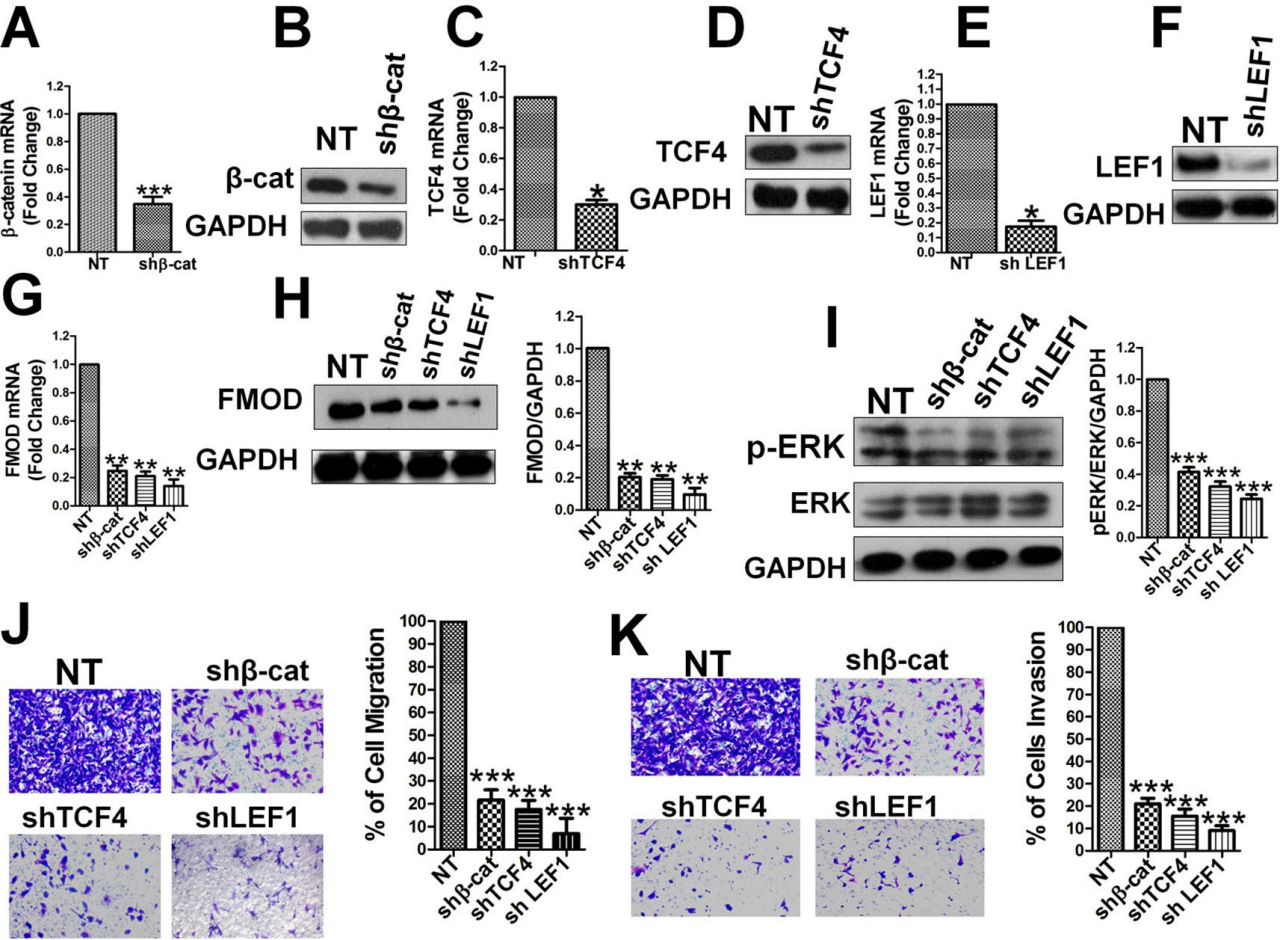
β-catenin, TCF4, and LEF1 are essential for FMOD expression, ERK activity, and BCCMI. The effects of depleting β-catenin, TCF4 or LEF1 by shRNA knockdown were tested in MDA-MB-231 cells. The knockdown and other treatments and analysis were conducted as described in **Figure 3**. **(A)** and **(B)** Knockdown of β-catenin mRNA **(A)** and protein **(B)** was achieved. **(C)** and **(D)** Knockdown of TCF4 mRNA **(C)** and protein **(D)** was achieved. **(E)** and **(F)** Knockdown of LEF1 mRNA **(E)** and protein **(F)** were achieved. **(G)** and (H) Knockdown of β-catenin, TFC4 or LEF1 led to decrease in FMOD expression at mRNA **(G)** and protein **(H)** level. **(I)** Knockdown of β-catenin, TFC4 or LEF1 led to the suppression of ERK activation. **(J)** and **(K)** Knockdown of β-catenin, TFC4 or LEF1 led to inhibition of cell migration **(J)** and invasion **(K)**. Data is shown as mean ± SD of 3 to 8 independent experiments. Student two-tailed t-test was used for statistical analysis (*P 0.05, **P 0.01, and ***P 0.001).

Subsequently, we tested whether β-catenin, TCF4, and LEF1 directly regulate endogenous FMOD gene expression at the mRNA and protein levels. Lentivirus-mediated shRNA was used to knock-down β-catenin, TCF4, and LEF1 individually, and the specificity and efficiency of the RNAi was confirmed by qPCR and western blot. β-catenin mRNA (**Figure 5A**) and protein (**Figure 5B**) levels were largely knocked-down in MDA-MB-231 cells, as were TCF4 mRNA (**Figure 5C**), TCF4 protein (**Figure 5D**), LEF1 mRNA (**Figure 5E**) and LEF1 protein (**Figure 5F**) levels. FMOD mRNA (**Figure 5G**) and protein (**Figure 5H**) levels were significantly reduced in these MDA-MB-231 cells where β-catenin, TCF4, and LEF1 were individually knocked down, indicating that β-catenin, TCF4, and LEF1 are each required for FMOD transcription. Taken together, these results demonstrate that β-catenin is a transcriptional coactivator that associates with transcription factors TCF4 and LEF1 to form a complex that regulates FMOD transcription. Using lentivirus-mediated shRNA targeted for β-catenin, TCF4, and LEF1, we investigated the relation between endogenous β-catenin, TCF4, LEF1, ERK1/2 activity, and breast cancer cell migration and invasion. Knockdown of β-catenin, TCF4 or LEF1 significantly down-regulated active p-ERK1/2 (**Figure 5I**). Transwell experiments showed a dramatic inhibition of cell migration (**Figure 5J**) and invasion (**Figure 5K**) due to the reduction of β-catenin, TCF4, and LEF1 in the breast cancer cells. Moreover, re-expression of FMOD reversed the inhibitory effects caused by knocking down β-catenin, TCF4, and LEF1 expression on cell migration (**Figure S2A**), invasion (**Figure S2B**), and ERK phosphorylation (**Figure S2C**). These results demonstrate that β-catenin, TCF4, and LEF1 are crucial in controlling FMOD expression and hence for ERK activation and BCCMI.

### Aspirin Downregulates TCF4/LEF1 and β-Catenin Expression Which Is Self-Promoted by the Wnt/β-Catenin Pathway

**Figure 6:**
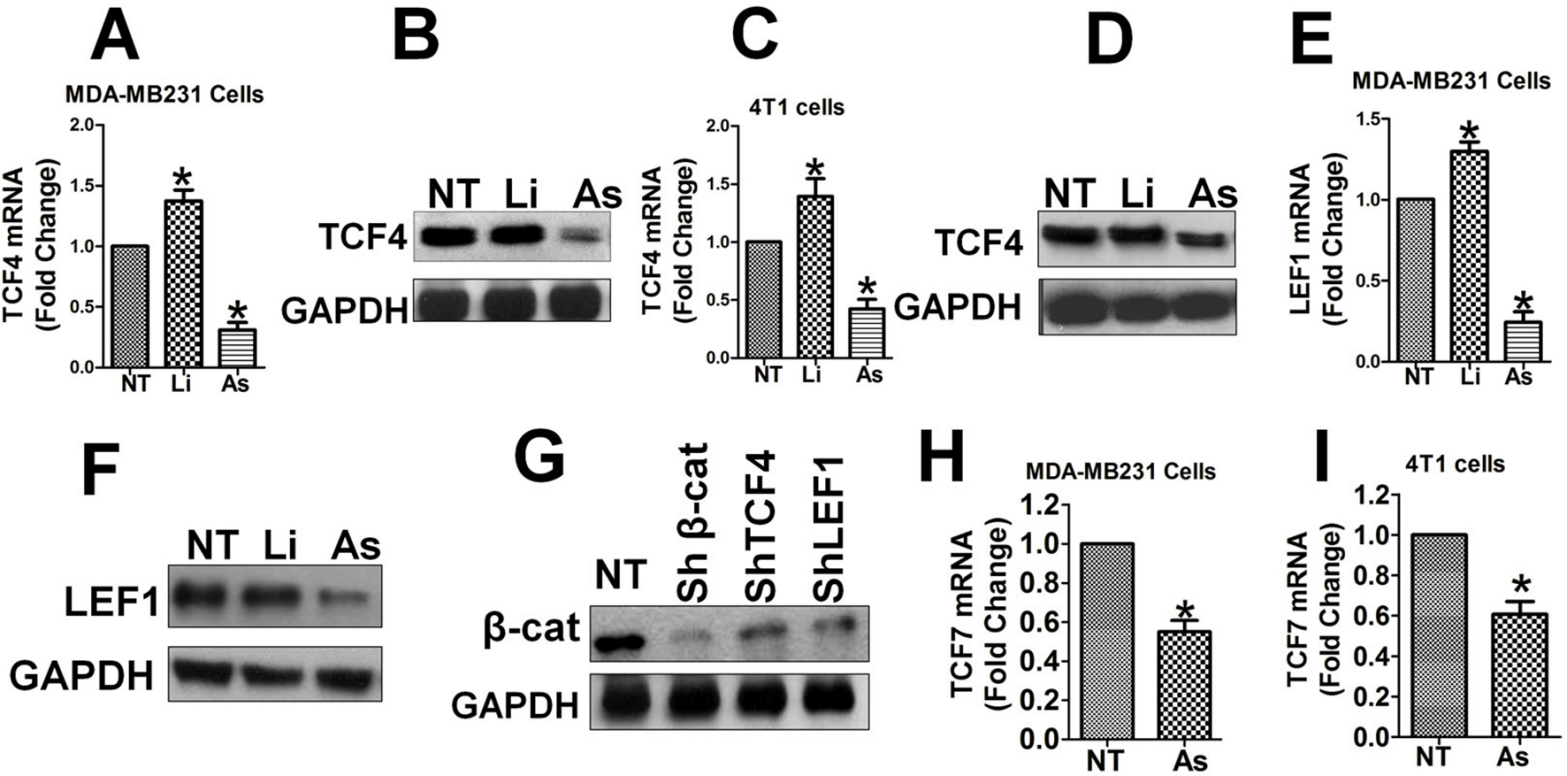
Aspirin downregulates TCF, LEF1 and β-catenin expression which is self-promoted by the Wnt/β-catenin pathway. The effects of Aspirin or LiCl on the transcription of TCF4, LEF1 andTCF7 **(A**–**F**, **H**, **I)**, and the effects of TCF4, LEF1 or β-catenin knockdown on the protein level of β-catenin **(G)** were tested in MDA-MB-231 cells **(A**, **B**, **E**, **F**, **G**, and **H)** and 4T1 cells **(C**, t**D**, and **I)**. Experiments and analysis were performed as described in **Figure 3**. **(A)** and **(B)** Expression level of TCF4 mRNA **(A)** and protein **(B)** in MDA-MB-231 cells. **(C)** and **(D)** Expression level of TCF4 mRNA **(C)** and protein **(D)** in 4T1 cells. **(E)** and **(F)** Expression level of LEF1 mRNA **(E)** and protein **(F)** in MDA-MB-231 cells. **(G)** Total β-catenin protein was markedly decreased by knockdown of TCF4, LEF1 or β-catenin in MDA-MB-231 cells. **(H)** Expression level of TCF7 mRNA in MDA-MB-231 cells. **(I)** Expression level of TCF7 mRNA in 4T1 cells. Data is shown as mean ± SD of 3 to 6 independent experiments. Student two-tailed t-test was used for statistical analysis (*P 0.05).

Subsequently, we investigated the effects of Aspirin and Wnt/β-catenin signaling on the expression levels of the β-catenin/TCF4/LEF1 complex. TCF4 mRNA and protein expression was upregulated by LiCl and down-regulated significantly by Aspirin in MDA-MB-231 cells (**Figures 6A, B**), as well as in 4T1 cells (**Figures 6C, D**). LEF1 mRNA and protein expression was also upregulated by LiCl and down-regulated significantly by Aspirin in MDA-MB-231 cells (**Figures 6E, F**). Additionally, TCF7 mRNA expression was significantly upregulated by LiCl and down-regulated by Aspirin in MDA-MB-231 (**Figure 6H**) and 4T1 cells (**Figure 6I**). This data suggests that the expression of the transcription factors TCF/LEF1 themselves is promoted by the Wnt/β-catenin pathway and inhibited by Aspirin. Besides, the level of total β-catenin protein was largely down-regulated in MDA-MB-231 cells in which TCF4, LEF1 or β-catenin were knocked down using lentiviral shRNA (**Figure 6G**), suggesting that the level of TCF4/LEF1 in turn positively impacts the level of β-catenin. These results indicate that through antagonizing Wnt/β-catenin signals, Aspirin has an inhibitory effect on the expression levels of the transcriptional factors TCF4 and LEF1, and also β-catenin, in addition to its inhibitory effect on the nuclear translocation of β-catenin (see below). Additionally, the results suggest that Aspirin modulates transcription of FMOD and BCCMI and also affects the expression level of the components of the β-catenin/TCF4/LEF1 complex, and the Wnt/β-catenin signaling pathway self-upregulates its components to amplify its signal through a positive feedback loop.

### Aspirin Impedes the Wnt/β-Catenin Pathway By Promoting β-Catenin Acetylation, Phosphorylation and Cytoplasmic Degradation and Inhibiting Its Nuclear Accumulation to Suppress FMOD Transcription

**Figure 7:**
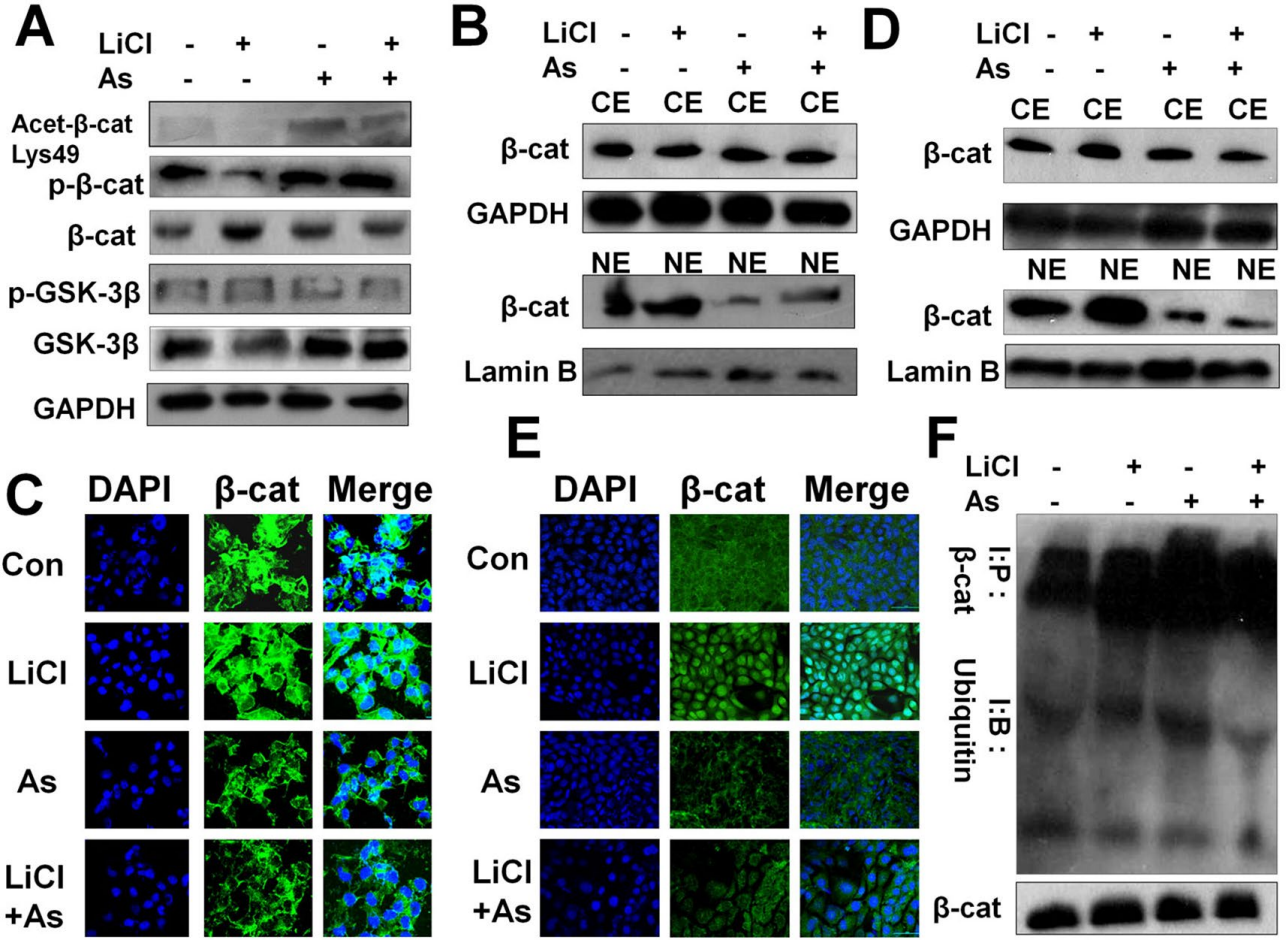
Aspirin impedes the Wnt/β-catenin pathway by promoting β-catenin acetylation, phosphorylation and cytoplasmic degradation, and inhibiting its nuclear accumulation and transcriptional activity. **(A)** Western blot of p-β-catenin, β-cateninm, p-GSK-3β, GSK-3β and acetylated β-catenin in MDA-MB-231 cells incubated with or without 10 mM LiCl for 6 h prior to treatment with or without 5 mM Aspirin for 24 h. **(B)** Western blot analysis of β-catenin protein distribution in nuclear extract (NE) and cytoplasmic fractions (CE) of MDA-MB-231 cells incubated with or without 10 mM LiCl for 6 h prior to treatment with 5 mM Aspirin for 24 h. **(C)** Immunofluorescent staining of MDA-MB-231 cells that were treated with or without Aspirin or LiCl. Cells were fixed and stained with a β-catenin antibody (green) and DAPI (blue), and imaged by confocal microscopy. **(D)** Same procedure performed as in **(B)** with the use of 4T1 cells. **(E)** Same procedure performed as in **(C)** with the use of 4T1 cells. **(F)** Western blot analysis of β-catenin and ubiquitinated β-catenin in MDA-MB-231 cells that were incubated with the indicated reagents for 24 h and subsequently treated with proteasome inhibitor benzyloxycarbonyl-leu-leu-leu-aldehyde (MG132) 25 µM for 5 h before harvest. Endogenous β-cat was immunoprecipitated with anti-β-catenin antibodies and Western blot was performed with anti-β-catenin or anti-ubiquitin antibodies.

We next investigated the mechanism whereby Aspirin attenuates Wnt/β-catenin signal-mediated FMOD gene transcription. In colon cancer, Aspirin has been reported to inhibit the canonical Wnt/β-catenin signaling pathway by inducing β-catenin phosphorylation (Dihlmann et al., 2003; Bos et al., 2006) resulting in the downregulation of nuclear β-catenin and TCF4 signaling. We found that Aspirin inhibits aberrant activation of the Wnt/β-catenin signaling pathway in triple-negative breast cancer MDA-MB-231 cells. Aspirin treatment resulted in a marked increase, while LiCl treatment resulted in a marked decrease, in phosphorylated β-catenin in the cell lysate (**Figure 7A**), which is consistent with previously published studies (Dihlmann et al., 2003; Ziegler et al., 2005; di Palma et al., 2006). As also shown in **Figure 7A**, Aspirin caused a marked increase in acetylated β-catenin (Lys-49) but a slight increase in GSK-3β, whereas LiCl caused a slight decrease in acetylated β-catenin but a marked decrease in GSK-3β. These results suggest that upstream of β-catenin, Aspirin exerts an inhibitory effect on the Wnt/β-catenin pathway mainly through promoting acetylation (inhibiting HDAC6, see below) and phosphorylation of β-catenin, while LiCl exerts a promotive effect on the pathway mainly through reducing active GSK-3β.

β-catenin activity is known to depend on its subcellular localization which is determined by its phosphorylation status. As the phosphorylation of β-catenin was induced by Aspirin in MDA-MB-231 cells (**Figure 7A**), we subsequently determined the subcellular distribution of β-catenin in MDA-MB-231 cells and 4T1 cells after being treated with LiCl or Aspirin. We observed that phospho-β-catenin levels were uniformly amplified in the cytoplasmic fractions and reduced in the nuclear fractions of MDA-MB-231 (**Figure 7B**) and 4T1 (**Figure 7D**) cells by Aspirin, while LiCl had the opposite effect.

We then examined the subcellular localization of β-catenin using immunofluorescent staining and imaging. It was noted that in both MDA-MB-231 (**Figure 7C**) and 4T1 cells (**Figure 7E**), contrary to LiCl, Aspirin caused a substantial reduction in nuclear β-catenin accompanied by an increase in cytoplasmic β-catenin protein levels, which altogether suggests that β-catenin may be degraded in the cytoplasm. Indeed, following cell treatment with Aspirin, β-catenin was significantly polyubiquitinated (**Figure 7F**) suggesting that β-catenin is degraded in the cytoplasm. These results indicate that Aspirin enhances β-catenin phosphorylation and cytoplasmic localization and degradation to suppress Wnt/β-catenin pathway mediated FMOD transcription.

### Aspirin, Like HDAC6 Inhibitor TSA, Inhibit HDAC6 Activity to Enhance β-Catenin Acetylation and Phosphorylation Resulting in Suppression of FMOD Expression

**Figure 8:**
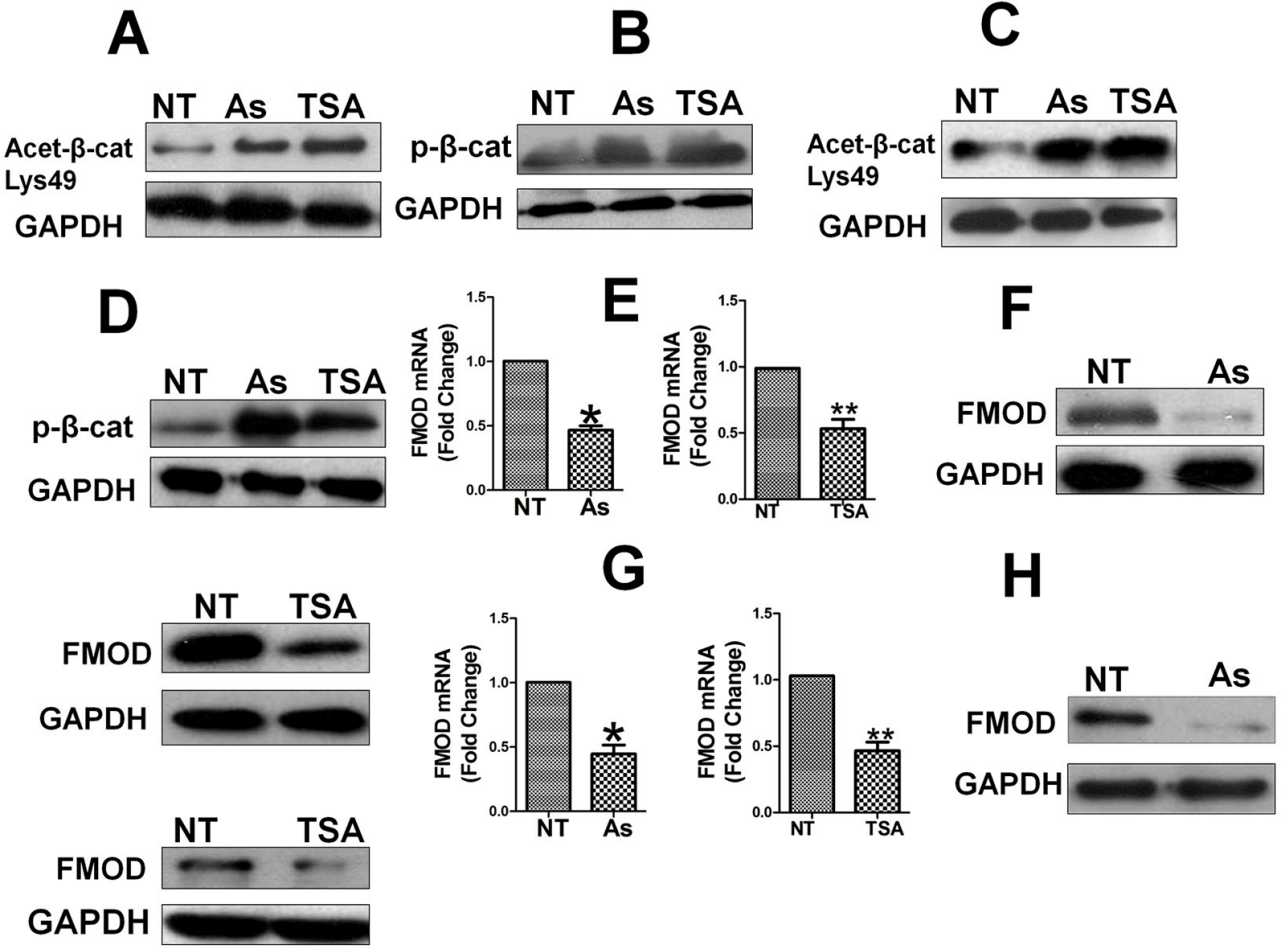
Aspirin, like HDAC6 inhibitor TSA, increase β-catenin acetylation and phosphorylation, leading to suppression of FMOD expression. Cells were treated with or without Aspirin at 5 mM or HDAC6 inhibitor TSA at 500 nM for 24 h. **(A)** and **(B)** Westen blot analysis of Acetylated β-catenin and phosphorylated β-catenin protein levels in MDA-MB-231 cells treated with Aspirin or TSA. **(C)** and **(D)** Western blot analysis of Acetylated β-catenin and phosphorylated β-catenin protein levels in 4T1 cells treated with Aspirin or TSA. **(E)** Quantification of FMOD mRNA levels in MDA-MB-231 cells treated with Aspirin and TSA. **(F)** Western blot analysis of FMOD protein levels in MDA-MB-231 cells treated with Aspirin and TSA. **(G)** Quantification of FMOD mRNA levels in 4T1 cells treated with Aspirin or TSA. **(H)** Western blot analysis of FMOD protein levels in 4T1 cells treated with Aspirin and TSA. P values are based on Student’s t-test (*P < 0.05 and **P < 0.01).

EGF-induced nuclear localization of β-catenin has been shown to be positively regulated by HDAC6 dependent deacetylation, as HDAC6 deacetylates β-catenin and inhibits its phosphorylation leading to its nuclear translocation, while HDAC6 inactivation enhances β-catenin acetylation and phosphorylation and blocks its nuclear translocation (Li et al., 2008). Having observed in our study that Aspirin promotes β-catenin phosphorylation and blocks its nuclear translocation, we hypothesized that HDAC6 may mediate the effects of Aspirin on the Wnt/β-catenin pathway and FMOD expression, i.e. Aspirin inhibits HDAC6 activity causing the acetylation of β-catenin to increase, which facilitates β-catenin phosphorylation and cytoplasmic degradation. To test this hypothesis, MDA-MB-231 and 4T1 cells were treated with Aspirin or TSA, a specific HDAC6 inhibitor, and the level of acetylated β-catenin was detected to reflect the activity of HDAC6, levels of β-catenin phosphorylation and FMOD expression was determined. As was partly shown with Aspirin in **Figure 7A**, Aspirin and TSA each caused a marked increase in acetylated β-catenin (**Figure 8A**) and phosphorylated β-catenin (**Figure 8B**) in MDA-MB-231 cells; and the same effects were observed in 4T1 cells (**Figures 8C, D**). As expected, Aspirin treatment resulted in a marked reduction of FMOD mRNA (**Figure 8E**, left) and protein (**Figure 8F**, left) in MDA-MB-231 cells, and in 4T1 cells (**Figures 8G, H**, left), as was also shown in **Figures 2A–D**; and likewise, TSA caused a marked reduction of FMOD mRNA (**Figure 8E**, right) and protein (**Figure 8F**, right) in MDA-MB-231 cells, and in 4T1 cells (**Figures 8G, H**, right).

These results indicate that having the same effects as TSA, Aspirin inhibits HDAC6 activity and HDAC6 mediates Aspirin’s inhibition of β-catenin, and HDAC6 evidently is a key target of Aspirin and a key molecular link between Aspirin and the Wnt/β-catenin pathway. Consequently, HDAC6 plays an important role in the regulation of the Wnt/β-catenin pathway-mediated nuclear translocation of β-catenin and expression of FMOD which leads to BCCMI.

### In BC Mouse Model, FMOD and β-Catenin Are Co-Overexpressed Highly and Sensitive to Aspirin Which Inhibits FMOD Expression and ERK Activation While Enhancing β-Catenin Phosphorylation and Preventing Its Nuclear Accumulation; and Per Clinical Databases, FMOD Expression Is Elevated in Invasive BC Patients Which Correlates With Worse Prognosis

**Figure 9:**
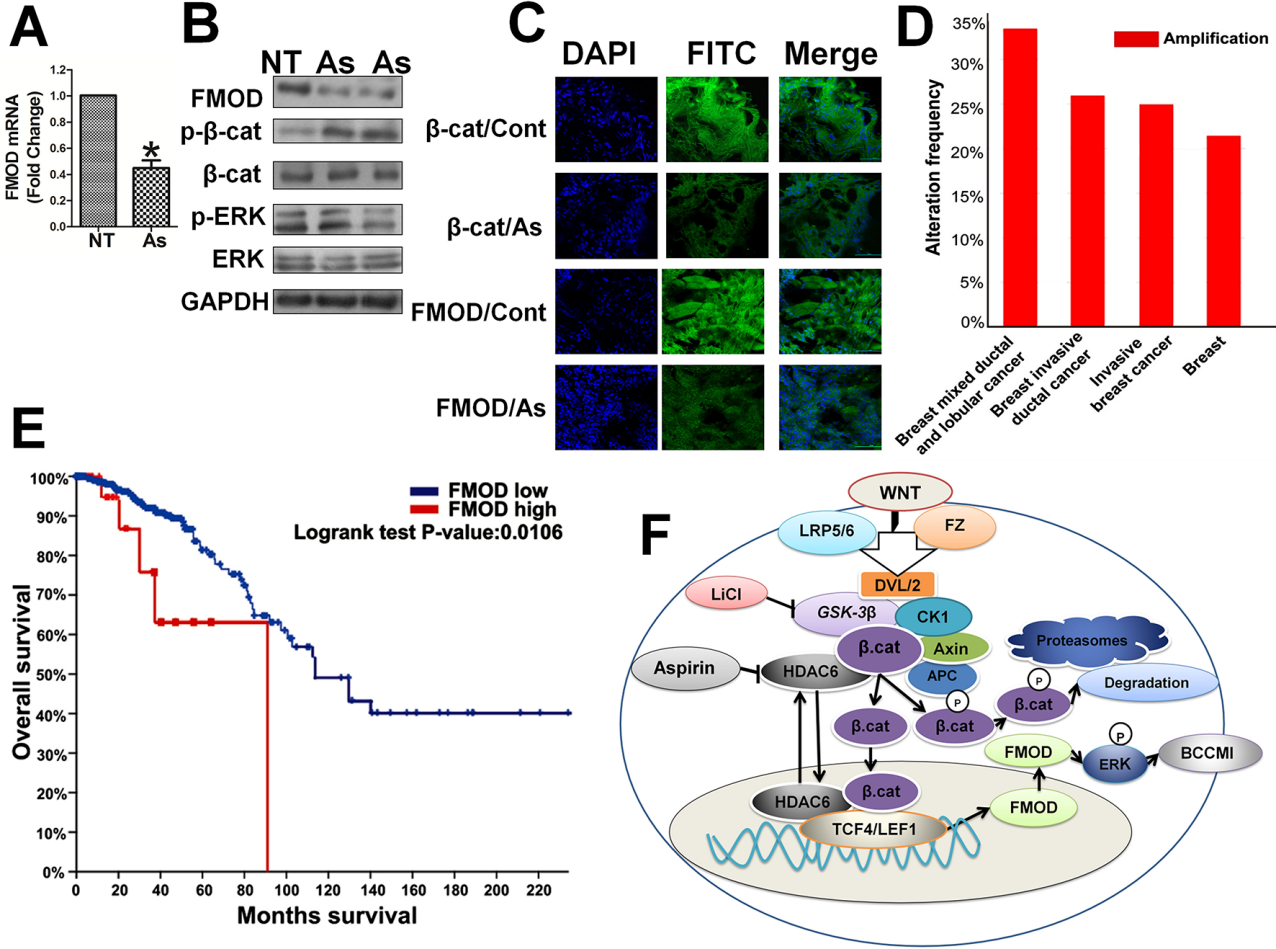
In human breast invasive tumor xenografts in mice, FMOD and β-catenin are highly co-expressed and sensitive to Aspirin which inhibits FMOD expression and ERK activation while enhancing β-catenin phosphorylation for cytoplasmic degradation and preventing its nuclear accumulation. In clinical data, elevated expression of FMOD occurs at a high frequency in invasive breast tumors and is associated with poor prognosis. **(A)** FMOD mRNA expression assayed by real-time RT-PCR in MDA-MB-231 cell tumor xenografts in female BALB/c nude mice treated with either PBS or 75 mg/kg Aspirin injections for 7 days. **(B)** Western blot analysis of FMOD, p-β-cat, β-cat, p-ERK and ERK protein expression levels in cell lysates from control (PBS treated) or Aspirin-treated mice. **(C)** Immunohistochemistry to show FMOD and β-catenin protein cytoplasmic and nuclear localization in MDA-MB-231 xenografts in control (PBS treated) or Aspirin-treated mice. Data are shown as the mean ± SD of three independent experiments. P values are based on Student’s t-test (*P 0.05). **(D)** The expression level of FMOD in breast cancer tissues of different breast cancer subtypes with those in normal tissues. Data from METABRIC, Nature 2012 and Nature Commun 2016. **(E)** Kaplan-Meier survival analysis and Log-rank test displayed overall survival of breast cancer patients with FMOD positive (n = 28) vs. FMOD negative (n = 454). Data from Breast Invasive Carcinoma (TCGA, Nature 2012). **(F)** Model of Aspirin and Wnt/β-catenin signaling pathway regulating fibromodulin (FMOD) transcription and cancer cell motility and invasiveness.

Thereafter, we further investigated the relation between FMOD and β-catenin and the effects of Aspirin *in vivo*. In the tumor xenografts in mice induced with human breast cancer MDA-MB-231 cells, expression of FMOD and β-catenin was elevated, Aspirin treatment greatly reduced FMOD mRNA expression (**Figure 9A**), β-catenin phosphorylation was increased, and p-ERK/ERK1/2 protein levels were reduced (**Figure 9B**). Immunohistochemical staining and imaging revealed that both β-catenin and FMOD were expressed at high levels in the invasive tumors, and Aspirin treatment decreased the total protein levels and also the nuclear distribution of β-catenin (**Figure 9C**). Moreover, analysis of clinical data from The Cancer Genome Atlas (TCGA) database indicated that FMOD is significantly upregulated in tumor samples including breast invasive ductal cancer and breast invasive lobular cancer (**Figure 9D**).Univariate analysis with Kaplan-Meier estimates revealed the specimens with FMOD positive staining were more likely to have an unfavorable survival when compared with the FMOD low group (p = 0.0106, **Figure 9E**). These animal model studies and patient clinical data analyses further support our *in vitro* results that illustrate the relationships in breast cancer between FMOD, β-catenin and Aspirin through the Wnt/β-catenin signaling pathway, and establish physiol-pathological significance of the regulation of FMOD expression by the Wnt/β-catenin pathway and the effects of Aspirin in the intervention of BCCMI.

## Discussion

In the present study, we demonstrate that in human and murine metastatic breast cancer cell lines, FMOD plays an essential role to promote BCCMI *via* activating ERK. Expression of FMOD, and hence BCCMI, are positively regulated by the Wnt/β-catenin signaling pathway, in which the β-catenin/TCF4/LEF1 transcriptional complex binds to the FMOD promoter at its TCF4-binding sites to transcribe FMOD. Meanwhile, the Wnt/β-catenin pathway upregulates expression of its own components TCF4/LEF1/β-catenin, forming positive feedback loops to amplify its signal output. Aspirin inhibits BCCMI through suppression of Wnt/β-catenin pathway-mediated FMOD expression. HDAC6 serves as a primary target molecule of Aspirin action linking the drug to the Wnt/β-catenin pathway, FMOD transcription and to BCCMI, i.e. Aspirin directly inhibits HDAC6 to enhance β-catenin acetylation, phosphorylation and cytoplasmic degradation, leading to suppression of the signaling pathway, FMOD expression and consequently BCCMI. Moreover, these molecular and cellular findings are further confirmed by results of *in vivo* investigations in human breast cancer mouse xenografts, and supported by analysis of clinical data from databases. Our present findings reveal important roles and inter-relationships of the ECM component FMOD, the Wnt/β-catenin signaling pathway and the NSAID drug Aspirin in BCCMI, which is summarized by a schematic model depicting Aspirin and the Wnt/β-catenin signaling pathway regulating fibromodulin (FMOD) expression and cancer metastasis (**Figure 9F**).

As an ECM protein, FMOD could be expected to play important roles in cancer, but there have been few studies on its involvement in cancer development in general, and essentially none reported in breast cancer metastasis in particular. A short study using overexpression of FMOD in murine mammary carcinoma cell line 4T1 suggested that FMOD overexpression down regulates NF-κB and TGF-β1, implying that FMOD may inhibit cell migration which depends on the NF-κB and TGF-β1 signaling pathways (Dawoody Nejad et al., 2017). In contrast, a recent more comprehensive study on FMOD in glioblastoma (GBM) indicated that FMOD is required for glioma cell migration, and that FMOD induces glioma cell migration induced by FMOD occurred *via* the activation of the FAK-Src-Rho-ROCK signaling pathway (Mondal et al., 2017). In one of our other studies (unpublished), FMOD was knocked out using the CRISPR/Cas9 system and it was observed that high expression of FMOD promoted breast cancer development and progression. Therefore, in this study, we compared the expression of FMOD in wild type breast cancer cells with breast cancer cells that had undergone lentiviral FMOD knockdown although it should be noted that a limitation of our study is the lack of scramble shRNA to act as a negative control. Our present study shows for the first time that FMOD is essential for breast cancer cell migration and invasion, which indicates that FMOD is likely a pro-metastatic factor in various cancers. We find that FMOD activates ERK to promote BCCMI, which is reminiscent of hyaluronan mediated motility receptor (RHAMM) promoting BCCMI *via* activation of ERK (Wang et al., 2014b), and undergoes the important part that ERK plays in cancer metastasis. Our study also suggests that, to facilitate BCCMI, FMOD mediates actin cytoskeleton assembly, which might additionally involve activating the FAK-Src-Rho-ROCK signaling pathway.

Elevated expression of FMOD has been associated with several types of cancer (Mayr et al., 2005; Reyes et al., 2016), and differential expression of FMOD has been observed in other pathophysiological conditions as well. Yet little was known about the molecular and cellular mechanisms by which FMOD expression is regulated, and only one study has been reported which suggested that FMOD transcription in glioma cells is positively regulated by the TGF-β1/SMAD2 pathway by an epigenetic mechanism (Mondal et al., 2017). We find for the first time that in breast cancer cells FMOD expression is regulated positively by the Wnt/β-catenin signaling pathway, and FMOD is transcribed by the β-catenin/TCF4/LEF1 transcription complex which binds to the FMOD promoter at specific sites. Thus our study identifies a cellular signaling pathway and the transcriptional factors and co-activator controlling FMOD expression, and establishes FMOD as a new key target gene of Wnt/β-catenin signaling pathway, and also as a downstream target of Aspirin inhibition as Aspirin acts on the Wnt/β-catenin pathway to suppress transcription of FMOD. Additionally, our results indicate that this FMOD transcriptional mechanism and its control by Wnt/β-catenin signaling also occurs in non-malignant HEK 293T cells, and thus may present widely in various tissues and tumors, although it remains to be determined how the Wnt/β-catenin mediated transcriptional mechanism is related to the TGF-β1/SMAD2 mediated epigenetic mechanism in regulating FMOD expression.

The Wnt/β-catenin signaling pathway has been extensively studied and its importance is well recognized. Our present study expands the functional roles and offers new insights into the operational mechanistics of the Wnt/β-catenin pathway. Our results confirm and extend the involvement of Wnt/β-catenin pathway in various tumors (Jansson et al., 2005; Iwai et al., 2010; Anastas and Moon, 2013; Dey et al., 2013) by demonstrating the crucial role of the Wnt/β-catenin pathway in promoting breast cancer metastasis *via* regulating FMOD transcription. Significantly, our results for the first time add the ECM component FMOD to the list of target genes of the Wnt/β-catenin pathway, where FMOD-mediated ERK activation is a path through which Wnt/β-catenin signaling facilitates cancer metastasis, thus linking Wnt/β-catenin signaling to the ECM, to ERK and to cancer metastasis. Moreover, we find that the Wnt/β-catenin signaling pathway upregulates expression of its own core components, β-catenin/TCF4/LEF1, to amplify its signal output, revealing a positive feedback regulatory mechanism. Several positive feedback loops within or involving the Wnt/β-catenin signaling pathway have been described (Kang et al., 2010; Zhu et al., 2013; Wu et al., 2016; Xu et al., 2018). Interestingly, we show that HDAC6, a deacetylase primarily localized in the cytoplasm, negatively regulates the acetylation and phosphorylation of β-catenin, i.e. positively regulates Wnt/β-catenin signaling, in agreement with previous reports (Li et al., 2008; Wang et al., 2014a) but in contrary to another study in hepatocellular carcinoma cells (Yin et al., 2018); and we find that HDAC6 is critical for the β-catenin signaling pathway to promote FMOD expression, and reveal HDAC6 as a component and regulator of the canonical Wnt/β-catenin pathway right upstream of β-catenin, and also as a direct target of Aspirin inhibition of this pathway and breast cancer (see below).

There have been many studies over the past decades about Aspirin in various cancers to investigate the anticancer effects of Aspirin as a chemopreventive or therapeutic agent (Cuzick et al., 2009; Langley et al., 2011), which revealed a number of different mechanisms and targets of Aspirin action. Aspirin was reported to down-regulate Wnt/β-catenin signaling by enhancing β-catenin phosphorylation levels in colorectal cancer cells (Dihlmann et al., 2003); to induce apoptosis in mesenchymal stem cells (MSCs) *via* the Wnt/β-catenin signaling pathway (Deng et al., 2009); and to inhibit glioma cell proliferation and invasive ability, and induce apoptosis *via* the β-catenin/TCF4 signaling pathway (Lan et al., 2011). In contrast, Aspirin was reported to inhibit breast tumor cell growth, EMT and migration *via* the TGF-β/SMAD4 signaling pathway (Maity et al., 2015); and to exert anti-tumor effects through other pathways such as the cyclooxygenase (COX) pathway and the NF-ĸB pathway in various cancers (Bilani et al., 2017; Chen and Holmes, 2017). Our present study clearly shows that Aspirin inhibits breast cancer cell migration and invasion *via* attenuating the Wnt/β-catenin signaling pathway to suppress transcription of FMOD, a newly identified target gene of the pathway, which establishes Wnt/β-catenin signaling pathway-mediated FMOD expression as a mechanism underlying the anti-metastasis effects of Aspirin in breast cancer.

On the other hand, previous studies have shown or implicated COX-2 (Williams et al., 1997) and other cellular components as targets of Aspirin’s anti-tumor effects; and protein phosphatase 2A (PP2A) was suggested to mediate the effect of Aspirin on the Wnt/β-catenin pathway in colorectal cancer cells (Bos et al., 2006). Our present study shows for the first time that Aspirin inhibits HDAC6 leading to increased acetylation and phosphorylation of β-catenin and hence decreased FMOD expression and BCCMI, i.e. HDAC6 is a direct target of Aspirin action that mediates the inhibitory effects of Aspirin on the Wnt/β-catenin pathway, and consequently on downstream FMOD, ERK and eventually cell migration and invasion in breast cancer, which identifies HDAC6 as a new target molecule of Aspirin action. In a sense, the components of the Wnt/β-catenin pathway downstream of HDAC6 such as β-catenin, FMOD and ERK are downstream targets of Aspirin effects. A latest study shows that Aspirin can cause acetylation of cGAS and inhibit immune responses mediated by the cGAS-STING pathway (Dai et al., 2019), underscoring the importance of further investigation into Aspirin’s effects and action mechanisms and generating renewed interest in this research subject. Further work will be needed to elucidate how the different mechanisms, pathways and targets of Aspirin action are related to one another, and how the components within a pathway are related to one another in cancers as well as other physiopathological conditions (Gala and Chan, 2015; Bilani et al., 2017; Chen and Holmes, 2017).

In summary, our present study establishes an important role of FMOD, and a specific mechanism of the Wnt/β-catenin signaling pathway regulating FMOD transcription in breast cancer; and identifies action targets and mechanism of Aspirin inhibition of Wnt/β-catenin pathway-mediated FMOD expression and breast cancer metastasis. As schematically illustrated in **Figure 9F**, our present results show that when the Wnt/β-catenin pathway is activated, β-catenin translocates into the nucleus where it forms a transcription complex with TCF4 and LEF1, which then promotes FMOD transcription and subsequent ERK activation leading to breast cancer cell migration and invasion. Furthermore, Aspirin inhibits breast cancer metastasis by suppressing FMOD expression mediated by the Wnt/β-catenin pathway, in which Aspirin suppresses FMOD transcription by attenuating the Wnt/β-catenin pathway, *via* inhibiting HDAC6 deacetylation of β-catenin to promote β-catenin phosphorylation, ubiquitination and cytoplasmic degradation. Thus our present study identifies the molecular and cellular mechanisms by which FMOD expression and BCCMI are regulated, and through which Aspirin exerts anti-metastatic effects on breast cancer by downregulating the HDAC6-β-catenin-FMOD-ERK axis. These unveil a manner by which Aspirin’s anticancer effects are tied to the regulation of expression of an important ECM component, FMOD. These insights are of significance and have clinical and therapeutic potential as our findings add FMOD to the list of β-catenin/TCF4 target genes, unveil a model by which aberrant Wnt/β-catenin signaling is involved in breast cancer progression, and indicate FMOD as well as HDAC6 as new potential targets and biomarkers for breast cancer therapy, which in turn could help guide the development and use of Aspirin as a cancer therapeutic.

## Significance Statement

Little was known about fibromodulin (FMOD) expression regulation and its role in cancer progression, or about the action targets and mechanisms of the anticancer benefits of Aspirin. We find that FMOD is essential in promoting breast cancer metastasis, FMOD expression is regulated positively by the Wnt/β-catenin signaling pathway, wherein the β-catenin/TCF4/LEF1 complex binds to the FMOD promoter for its transcription, and Aspirin inhibits breast cancer metastasis by attenuating the Wnt/β-catenin pathway and suppressing FMOD expression, evidently *via* inhibiting HDAC6 deacetylation of β-catenin.

Our findings identify a critical role of FMOD in cancer metastasis, reveal a mechanism regulating FMOD transcription and impacting tumor metastasis, unveil action targets and mechanism for the anticancer activity of Aspirin, and expand the understanding of the Wnt/β-catenin pathway and tumor metastasis, which are valuable for the development of cancer therapeutics.

## Data Availability Statement

The raw data supporting the conclusions of this manuscript will be made available by the authors, without undue reservation, to any qualified researcher.

## Ethics Statement

All the animal studies were carried out according to the protocol after receiving approval from the Administrative Committee on Animal Research of the Graduate School at Shenzhen, Tsinghua University. Written informed consent was obtained from the owners for the participation of their animals in this study.

## Author Contributions

LH and FK conceptualized and designed the research. FK conducted most of the the experiments and data analysis with the participation and assistance of XD, KL, YW, CS, HT, and HC. FK, NO-T, and LH drafted and revised the paper.

## Funding

This work was supported with funding awarded to LH from China MOST National Key Basic Research 973 Program (2005CCA03500), China NSFC (30570960, 30671034, 81641051, 81872368) Guangdong Province NSF (05010197), Shenzhen Municipal Science & Technology Programs and the Program for Building Shenzhen and State Key Laboratories (JCYJ20130402145002438, ZDSYS20140509172959975, GJHZ20140416153844269, JCYJ20140418112611757, JCYJ20180508152130899; JCYJ20180508153013853, GuoKeFaJi [2018]38), and Shenzhen Development and Reform Commission Discipline Development Project [2017]1434.

## Conflict of Interest

The authors declare that the research was conducted in the absence of any commercial or financial relationships that could be construed as a potential conflict of interest.

## References

[B1] Anastas J. N. Moon R. T. (2013). WNT signalling pathways as therapeutic targets in cancer. Nat. Rev. Cancer 13, 11–26. 10.1038/nrc3419 23258168

[B2] Bilani N. Bahmad H. Abou-Kheir W. (2017). Prostate Cancer and Aspirin Use: Synopsis of the Proposed Molecular Mechanisms. Front. Pharmacol. 8, 145. 10.3389/fphar.2017.00145 28377721 PMC5359278

[B3] Bonnans C. Chou J. Werb Z. (2014). Remodelling the extracellular matrix in development and disease. Nat. Rev. Mol. Cell Biol. 15, 786–801. 10.1038/nrm3904 25415508 PMC4316204

[B4] Bos C. L. Kodach L. L. van den Brink G. R. Diks S. H. van Santen M. M. Richel D. J. (2006). Effect of aspirin on the Wnt/beta-catenin pathway is mediated *via* protein phosphatase 2A. Oncogene 25, 6447–6456. 10.1038/sj.onc.1209658 16878161

[B5] Cao W. Zeng X. Liu G. Li Z. Zeng X. Wang L. (2015). Porphine functionalized nanoparticles of star-shaped poly(epsilon-caprolactone)-b-D-alpha-tocopheryl polyethylene glycol 1000 succinate biodegradable copolymer for chemophotodynamic therapy on cervical cancer. Acta Biomater. 26, 145–158. 10.1016/j.actbio.2015.08.016 26283167

[B6] Center for Disease Control and Prevention (CDC). (2017). United States Cancer Statistics: 1999–2014 Cancer Incidence and Mortality. CDC https://nccd.cdc.gov/uscs/.

[B7] Chen W. Y. Holmes M. D. (2017). Role of Aspirin in Breast Cancer Survival. Curr. Oncol. Rep. 19, 48. 10.1007/s11912-017-0605-6 28597105

[B8] Choudhury A. Derkow K. Daneshmanesh A. H. Mikaelsson E. Kiaii S. Kokhaei P. (2010). Silencing of ROR1 and FMOD with siRNA results in apoptosis of CLL cells. Br. J. Haematol. 151, 327–335. 10.1111/j.1365-2141.2010.08362.x 20813009

[B9] Cuzick J. Otto F. Baron J. A. Brown P. H. Burn J. Greenwald P. (2009). Aspirin and non-steroidal anti-inflammatory drugs for cancer prevention: an international consensus statement. Lancet Oncol. 10, 501–507. 10.1016/S1470-2045(09)70035-X 19410194

[B10] Dai J. Huang Y. J. He X. Zhao M. Wang X. Liu Z. S. (2019). Acetylation Blocks cGAS Activity and Inhibits Self-DNA-Induced Autoimmunity. Cell 176, 1447–1460.e1414. 10.1016/j.cell.2019.01.016 30799039 PMC8274936

[B11] Dawoody Nejad L. Biglari A. Annese T. Ribatti D. (2017). Recombinant fibromodulin and decorin effects on NF-kappaB and TGFbeta1 in the 4T1 breast cancer cell line. Oncol. Lett. 13, 4475–4480. 10.3892/ol.2017.5960 28599447 PMC5452964

[B12] Deng L. Hu S. Baydoun A. R. Chen J. Chen X. Cong X. (2009). Aspirin induces apoptosis in mesenchymal stem cells requiring Wnt/beta-catenin pathway. Cell Prolif. 42, 721–730. 10.1111/j.1365-2184.2009.00639.x 19706045 PMC6495846

[B13] Dey N. Barwick B. G. Moreno C. S. Ordanic-Kodani M. Chen Z. Oprea-Ilies G. (2013). Wnt signaling in triple negative breast cancer is associated with metastasis. BMC Cancer 13, 537. 10.1186/1471-2407-13-537 24209998 PMC4226307

[B14] di Palma A. Matarese G. Leone V. Di Matola T. Acquaviva F. Acquaviva A. M. (2006). Aspirin reduces the outcome of anticancer therapy in Meth A-bearing mice through activation of AKT-glycogen synthase kinase signaling. Mol. Cancer Ther. 5, 1318–1324. 10.1158/1535-7163.MCT-05-0473 16731765

[B15] Dihlmann S. Klein S. Doeberitz Mv M. (2003). Reduction of beta-catenin/T-cell transcription factor signaling by aspirin and indomethacin is caused by an increased stabilization of phosphorylated beta-catenin. Mol. Cancer Ther. 2, 509–516. 12813129

[B16] Drew D. A. Cao Y. Chan A. T. (2016). Aspirin and colorectal cancer: the promise of precision chemoprevention. Nat. Rev. Cancer 16, 173–186. 10.1038/nrc.2016.4 26868177 PMC6741347

[B17] Eckhardt B. L. Francis P. A. Parker B. S. Anderson R. L. (2012). Strategies for the discovery and development of therapies for metastatic breast cancer. Nat. Rev. Drug Discov. 11, 479–497. 10.1038/nrd2372 22653217

[B18] Edwards I. J. (2012). Proteoglycans in prostate cancer. Nat. Rev. Urol. 9, 196–206. 10.1038/nrurol.2012.19 22349653

[B19] Fong S. Itahana Y. Sumida T. Singh J. Coppe J. P. Liu Y. (2003). Id-1 as a molecular target in therapy for breast cancer cell invasion and metastasis. Proc. Natl. Acad. Sci. U. S. A. 100, 13543–13548. 10.1073/pnas.2230238100 14578451 PMC263850

[B20] Gala M. K. Chan A. T. (2015). Molecular pathways: aspirin and Wnt signaling-a molecularly targeted approach to cancer prevention and treatment. Clin. Cancer Res. 21, 1543–1548. 10.1158/1078-0432.CCR-14-0877 25501125 PMC4383688

[B21] Henderson L. J. Narasipura S. D. Adarichev V. Kashanchi F. Al-Harthi L. (2012). Identification of novel T cell factor 4 (TCF-4) binding sites on the HIV long terminal repeat which associate with TCF-4, beta-catenin, and SMAR1 to repress HIV transcription. J. Virol. 86, 9495–9503. 10.1128/JVI.00486-12 22674979 PMC3416155

[B22] Insua-Rodriguez J. Oskarsson T. (2016). The extracellular matrix in breast cancer. Adv. Drug Deliv. Rev. 97, 41–55. 10.1016/j.addr.2015.12.017 26743193

[B23] Iozzo R. V. Sanderson R. D. (2011). Proteoglycans in cancer biology, tumour microenvironment and angiogenesis. J. Cell Mol. Med. 15, 1013–1031. 10.1111/j.1582-4934.2010.01236.x 21155971 PMC3633488

[B24] Iwai S. Yonekawa A. Harada C. Hamada M. Katagiri W. Nakazawa M. (2010). Involvement of the Wnt-beta-catenin pathway in invasion and migration of oral squamous carcinoma cells. Int. J. Oncol. 37, 1095–1103. 10.3892/ijo_00000761 20878057

[B25] Jansson E. A. Are A. Greicius G. Kuo I. C. Kelly D. Arulampalam V. (2005). The Wnt/beta-catenin signaling pathway targets PPARgamma activity in colon cancer cells. Proc. Natl. Acad. Sci. U. S. A. 102, 1460–1465. 10.1073/pnas.0405928102 15665104 PMC547827

[B26] Kang D. W. Lee S. H. Yoon J. W. Park W. S. Choi K. Y. Min do S. (2010). Phospholipase D1 drives a positive feedback loop to reinforce the Wnt/beta-catenin/TCF signaling axis. Cancer Res. 70, 4233–4242. 10.1158/0008-5472.CAN-09-3470 20442281

[B27] Kim S. H. Turnbull J. Guimond S. (2011). Extracellular matrix and cell signalling: the dynamic cooperation of integrin, proteoglycan and growth factor receptor. J. Endocrinol. 209, 139–151. 10.1530/JOE-10-0377 21307119

[B28] Lambert A. W. Pattabiraman D. R. Weinberg R. A. (2017). Emerging Biological Principles of Metastasis. Cell 168, 670–691. 10.1016/j.cell.2016.11.037 28187288 PMC5308465

[B29] Lan F. Yue X. Han L. Yuan X. Shi Z. Huang K. (2011). Antitumor effect of aspirin in glioblastoma cells by modulation of beta-catenin/T-cell factor-mediated transcriptional activity. J. Neurosurg. 115, 780–788. 10.3171/2011.5.JNS113 21721879

[B30] Langley R. E. Burdett S. Tierney J. F. Cafferty F. Parmar M. K. Venning G. (2011). Aspirin and cancer: has aspirin been overlooked as an adjuvant therapy? Br. J. Cancer 105, 1107–1113. 10.1038/bjc.2011.289 21847126 PMC3208483

[B31] Larue L. Bellacosa A. (2005). Epithelial-mesenchymal transition in development and cancer: role of phosphatidylinositol 3’ kinase/AKT pathways. Oncogene 24, 7443–7454. 10.1038/sj.onc.1209091 16288291

[B32] Li Y. Zhang X. Polakiewicz R. D. Yao T. P. Comb M. J. (2008). HDAC6 is required for epidermal growth factor-induced beta-catenin nuclear localization. J. Biol. Chem. 283, 12686–12690. 10.1074/jbc.C700185200 18356165 PMC3762558

[B33] Liu Y. Cao W. Zhang B. Liu Y. Q. Wang Z. Y. Wu Y. P. (2013). The natural compound magnolol inhibits invasion and exhibits potential in human breast cancer therapy. Sci. Rep. 3, 3098. 10.1038/srep03098 24226295 PMC3827615

[B34] MacDonald B. T. Tamai K. He X. (2009). Wnt/beta-catenin signaling: components, mechanisms, and diseases. Dev. Cell 17, 9–26. 10.1016/j.devcel.2009.06.016 19619488 PMC2861485

[B35] Maity G. De A. Das A. Banerjee S. Sarkar S. Banerjee S. K. (2015). Aspirin blocks growth of breast tumor cells and tumor-initiating cells and induces reprogramming factors of mesenchymal to epithelial transition. Lab. Invest. 95, 702–717. 10.1038/labinvest.2015.49 25867761

[B36] Mayr C. Bund D. Schlee M. Moosmann A. Kofler D. M. Hallek M. (2005). Fibromodulin as a novel tumor-associated antigen (TAA) in chronic lymphocytic leukemia (CLL), which allows expansion of specific CD8+ autologous T lymphocytes. Blood 105, 1566–1573. 10.1182/blood-2004-04-1233 15471955

[B37] Mondal B. Patil V. Shwetha S. D. Sravani K. Hegde A. S. Arivazhagan A. (2017). Integrative functional genomic analysis identifies epigenetically regulated fibromodulin as an essential gene for glioma cell migration. Oncogene 36, 71–83. 10.1038/onc.2016.176 27212030

[B38] Nelson J. D. Denisenko O. Bomsztyk K. (2006). Protocol for the fast chromatin immunoprecipitation (ChIP) method. Nat. Protoc. 1, 179–185. 10.1038/nprot.2006.27 17406230

[B39] Oldberg A. Kalamajski S. Salnikov A. V. Stuhr L. Morgelin M. Reed R. K. (2007). Collagen-binding proteoglycan fibromodulin can determine stroma matrix structure and fluid balance in experimental carcinoma. Proc. Natl. Acad. Sci. U. S. A. 104, 13966–13971. 10.1073/pnas.0702014104 17715296 PMC1955775

[B40] Orford K. Crockett C. Jensen J. P. Weissman A. M. Byers S. W. (1997). Serine phosphorylation-regulated ubiquitination and degradation of beta-catenin. J. Biol. Chem. 272, 24735–24738. 10.1074/jbc.272.40.24735 9312064

[B41] Pietraszek-Gremplewicz K. Karamanou K. Niang A. Dauchez M. Belloy N. Maquart F. X. (2019). Small leucine-rich proteoglycans and matrix metalloproteinase-14: Key partners? Matrix Biol. 75-76, 271–285. 10.1016/j.matbio.2017.12.006 29253518

[B42] Reyes N. Benedetti I. Bettin A. Rebollo J. Geliebter J. (2016). The small leucine rich proteoglycan fibromodulin is overexpressed in human prostate epithelial cancer cell lines in culture and human prostate cancer tissue. Cancer Biomark 16, 191–202. 10.3233/CBM-150555 26600400 PMC13016539

[B43] Ring L. Neth P. Weber C. Steffens S. Faussner A. (2014). beta-Catenin-dependent pathway activation by both promiscuous “canonical” WNT3a-, and specific “noncanonical” WNT4- and WNT5a-FZD receptor combinations with strong differences in LRP5 and LRP6 dependency. Cell Signal 26, 260–267. 10.1016/j.cellsig.2013.11.021 24269653

[B44] Silva-Garcia O. Valdez-Alarcon J. J. Baizabal-Aguirre V. M. (2014). The Wnt/beta-catenin signaling pathway controls the inflammatory response in infections caused by pathogenic bacteria. Mediators Inflamm. 2014, 310183. 10.1155/2014/310183 25136145 PMC4127235

[B45] Tetsu O. McCormick F. (1999). Beta-catenin regulates expression of cyclin D1 in colon carcinoma cells. Nature 398, 422–426. 10.1038/18884 10201372

[B46] Wang S. H. Li N. Wei Y. Li Q. R. Yu Z. P. (2014a). beta-catenin deacetylation is essential for WNT-induced proliferation of breast cancer cells. Mol. Med. Rep. 9, 973–978. 10.3892/mmr.2014.1889 24401947

[B47] Wang Z. Wu Y. Wang H. Zhang Y. Mei L. Fang X. (2014b). .Interplay of mevalonate and Hippo pathways regulates RHAMM transcription *via* YAP to modulate breast cancer cell motility. Proc. Natl. Acad. Sci. U S. A. 111, E89–E98. 10.1073/pnas.1319190110 24367099 PMC3890879

[B48] Williams C. S. Smalley W. DuBois R. N. (1997). Aspirin use and potential mechanisms for colorectal cancer prevention. J. Clin. Invest. 100, 1325–1329. 10.1172/JCI119651 9294096 PMC508309

[B49] Wu C. Zhang H. F. Gupta N. Alshareef A. Wang Q. Huang Y. H. (2016). A positive feedback loop involving the Wnt/beta-catenin/MYC/Sox2 axis defines a highly tumorigenic cell subpopulation in ALK-positive anaplastic large cell lymphoma. J. Hematol. Oncol. 9, 120. 10.1186/s13045-016-0349-z 27821172 PMC5100098

[B50] Xu G. Wang Y. Li W. Cao Y. Xu J. Hu Z. (2018). COX-2 Forms Regulatory Loop with YAP to Promote Proliferation and Tumorigenesis of Hepatocellular Carcinoma Cells. Neoplasia 20, 324–334. 10.1016/j.neo.2017.12.004 29505957 PMC5909490

[B51] Yin Z. Xu W. Xu H. Zheng J. Gu Y. (2018). Overexpression of HDAC6 suppresses tumor cell proliferation and metastasis by inhibition of the canonical Wnt/beta-catenin signaling pathway in hepatocellular carcinoma. Oncol. Lett. 16, 7082–7090. 10.3892/ol.2018.9504 30546442 PMC6256338

[B52] Zhang J. Huang K. Shi Z. Zou J. Wang Y. Jia Z. (2011). High beta-catenin/Tcf-4 activity confers glioma progression *via* direct regulation of AKT2 gene expression. Neuro. Oncol. 13, 600–609. 10.1093/neuonc/nor034 21636708 PMC3107098

[B53] Zhu X. Morales F. C. Agarwal N. K. Dogruluk T. Gagea M. Georgescu M. M. (2013). Moesin is a glioma progression marker that induces proliferation and Wnt/beta-catenin pathway activation *via* interaction with CD44 . Cancer Res. 73, 1142–1155. 10.1158/0008-5472.CAN-12-1040 23221384

[B54] Ziegler S. Rohrs S. Tickenbrock L. Moroy T. Klein-Hitpass L. Vetter I. R. (2005). Novel target genes of the Wnt pathway and statistical insights into Wnt target promoter regulation. FEBS J. 272, 1600–1615. 10.1111/j.1742-4658.2005.04581.x 15794748

