# Supplementary material for: Wnt/β-Catenin Pathway-Regulated Fibromodulin Expression Is Crucial for Breast Cancer Metastasis and Inhibited by Aspirin
Source: Front Pharmacol. 2019 Nov 25;10:1308. doi: 10.3389/fphar.2019.01308 (PMC6886402; doi:10.3389/fphar.2019.01308)
Supplement: Supplementary file 1 [file DataSheet_1.pdf]

Supplemental Information

Wnt/β-Catenin Pathway-Regulated Fibromodulin Expression Is Crucial for Breast Cancer Metastasis and Inhibited by Aspirin

Fahim Ullah Khan[1,2], Nana Yaa Gyaama Owusu-Tieku[3], Xiaoyong Dai[3], Kewei Liu[1,2], Yanping Wu[1,2], Chunhui Sun[3], Laiqiang Huang[1,2,3,*].

Tsinghua University

Table 1. shRNA Sequence

Gene name	Sequence 5′-3′
**1. FMODshRNA1 (Human)-sense**	GATCCGCTCTTGAGCAGCTGTACATTCAAGAGATGTACAGCTGCTCAAGAGCTTTTTTGGAAA
**FMOD shRNA1 (Human)-anti-sense**	AGCTTTTCCAAAAAAGCTCTTGAGCAGCTGTACATCT CTTGAATGTACAGCTGCTCAAGAGCG
**2. FMODshRNA2 (Human)-sense**	GATCCAGCTCTTGAGCAGCTGTACTTC AAGAGAGTACAGCTGCTCAAGAGCTTTTTTTGGAAA
**FMOD shRNA2 (Human)-anti-sense**	AGCTTTTCCAAAAAAAGCTCTTGAGCAGCTGTAC TCTCTTGAAGTACAGCTGCTCAAGAGCTG
**3. β-catenin Human shRNA-sense**	GATCCTTGGAATGAGACTGCTGATTTC AAGAGAATCAGCAGTCTCATTCCAATTTTTTGGAAA
**β- catenin Human shRNA-anti- sense**	AGCTTTTCCAAAAAATTGGAATGAGACTGCTGATTCTCTT GAAATCAGCAGTCTCATTCCAAG
**4. TCF4 Human shRNA-sense**	ATCCTTCACCTCCTGTGAGCAGTTTCAAGAGAACTGCTCACAGGAGGTGAATTTTTTGGAAA
**TCF4 Human shRNA-anti- sense**	AGCTTTTCCAAAAAATTCACCTCCTGTGAGCAGTTCTCTTGAAACTGCTCACAGGAGGTGAAG
**5. LEF1 shRNA Human-sense**	GATCC GAT CTT CGC CGA GAT CAG T TT C AAG AGA ACT GAT CTC GGC GAA GAT C TTTTTTGGAA A
**LEF1 shRNA Human-anti-sense**	AGCTTTTCCAAAAAAGATCTTCGCCGAGATCAGTTCTCTTGAAACTGATCTCGGCGAAGATCG

Table 2. Primers Used in Cloning and for qPCR

	Gene name	5′-3′ Sense Primer	5′-3′ Anti-Sense Primer
1.	**FMOD (Human)**	ATGCAGTGGACCTCCCTCCT	TCAGATCTCGATGAGGCTGG
2.	**FMOD (Mouse)**	ATGCTTTGGAGGGCCTGGAG	TCAGATCTCGATGAGGTTGG
3.	**GAPDH (Human)**	CCAGAACATCATCCCTGCCTCTACT	GGTTTTTCTAGACGGCAGGTCAGGT
4.	**GAPDH (Mouse)**	TGGATTTGGACGCATTGGTC	TTTGCACTGGTACGTGTTGAT
5.	**TCF7 (Human)**	TGCAGCTATACCCAGGCTGG	CCTCGACCGCCTCTTCTTC
6.	**TCF7 (Mouse)**	CCCAGCTTTCTCCACTCTAC	TGTGAACTCCTTGCTTCTGG
7.	**LEF1(Human)**	CGACGCCAAAGGAACACTGACATC	GCACGCAGATATGGGGGAGAAA
8.	**LEF1(Mouse)**	GCAGCTATCAACCAGATCC	GATGTAGGCAGCTGTCATTC
9.	**N-Cadherin(Human)**	ATGTGCCGGATAGCGGGAGC	TACACCGTGCCGTCCTCGTC
10.	**β-catenin (Human)**	CAACTAAACAGGAAGGGATG	CACAGGTGACCACATTTATATC
11.	**TCF4 (Human)**	ATGGCAAATAGAGGAAGCGG	TGGAGAATAGATCGAAGCAAG

Table 3. Primers Used in Cloning

	Gene name	Sequence 5′-3′
1.	**Wnt-3a-sense**	AAATTAAGCTTATGGCCCCACTCGGATACTTC
	**Wnt-3a- anti-sense**	TATGGTACCCTTGCAGGTGTGCACGTCGTAGAC
2.	**FMOD-PGL3 -sense**	CGCTCGAGACTGATGAGCCCTCTTTCTCATCTATTC
	**FMOD-PGL3 -anti-sense**	CATGCCATGGCTTGAGGAGAGGACTGTGATTAGA
3.	**FMOD-PBOBI-sense**	TGCTCTAGAATGCAGTGGACCTCCCTCCTG
	**FMOD-PBOBI-anti-sense**	CCGCTCGAGGGAGAAGTTCACGACGTCCACC
