# Supplementary material for: Wnt/β-Catenin Pathway-Regulated Fibromodulin Expression Is Crucial for Breast Cancer Metastasis and Inhibited by Aspirin
Source: Front Pharmacol. 2019 Nov 25;10:1308. doi: 10.3389/fphar.2019.01308 (PMC6886402; doi:10.3389/fphar.2019.01308)
Supplement: Supplementary file 2 [file DataSheet_2.docx]

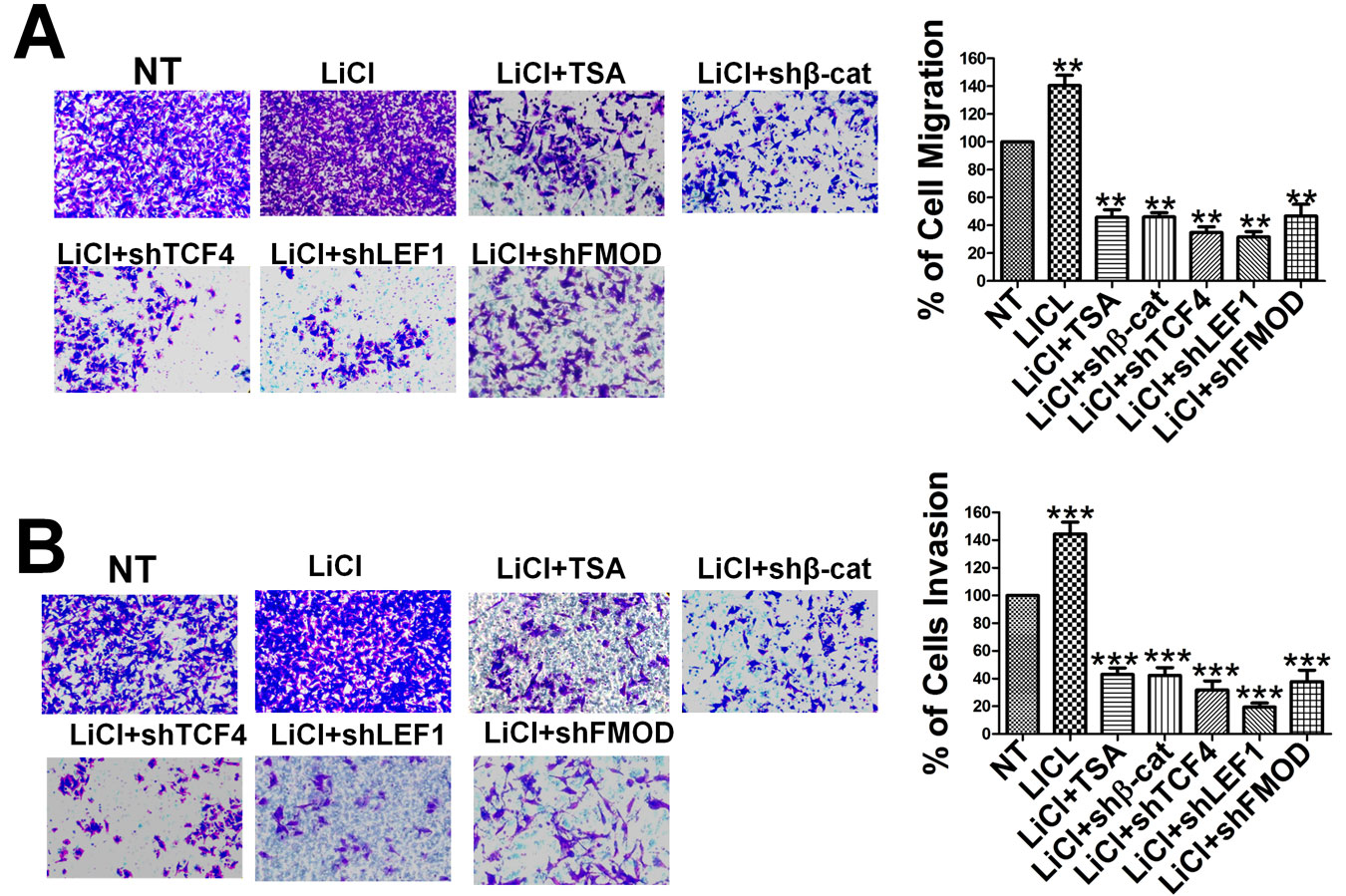


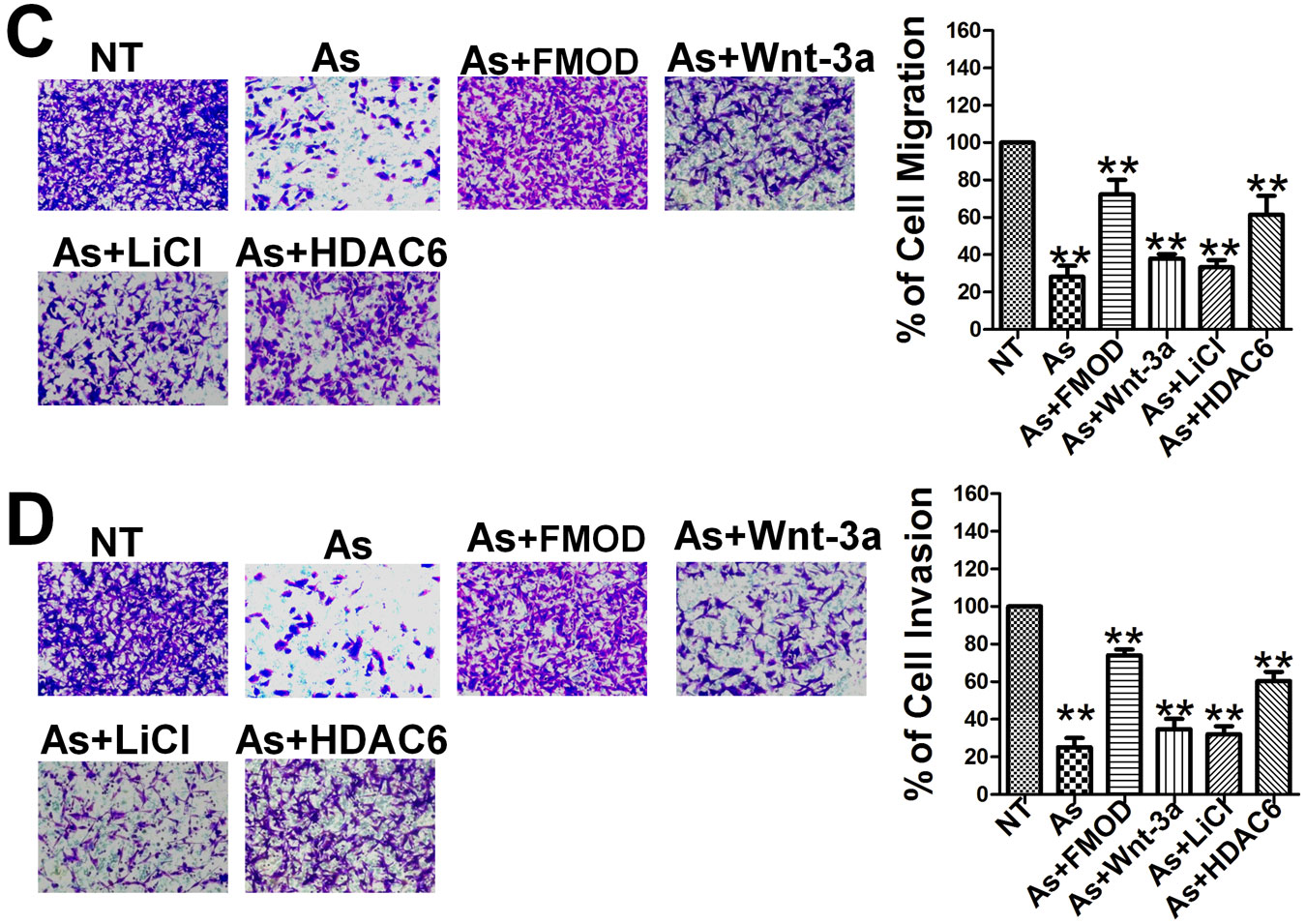


Figure S1. Enhancement of BCCMI by LiCl is nullified by knocking down FMOD or a component of β-cat/TCF4/LEF1 complex, or by inhibiting HDAC6 with TSA; and inhibition of BCCMI by Aspirin was reversed largely by overexprssing FMOD or HDAC6 but scarcely by LiCl or overexpressing Wnt-3a.

MDA-MB231 cells were treated with LiCl for 6h and then treated with or without shβ-cat, shTCF4, shLEF1 or shFMOD expression, or HDAC6 inhibitor TSA (A, B). The cells were treated with Aspirin for 24 h and then with or without FMOD, HDAC6 or Wnt-3a overexpression, or LiCl (C, D).

(A) and (B) Cell migration, Invasion, and quantification.

(C) and (D) Cell migration, invasion, and quantification.

Data are shown as mean ± SD of three independent experiments. Student two-tailed t-test was used for statistical analysis (*P < 0.01 and ***P < 0.001).


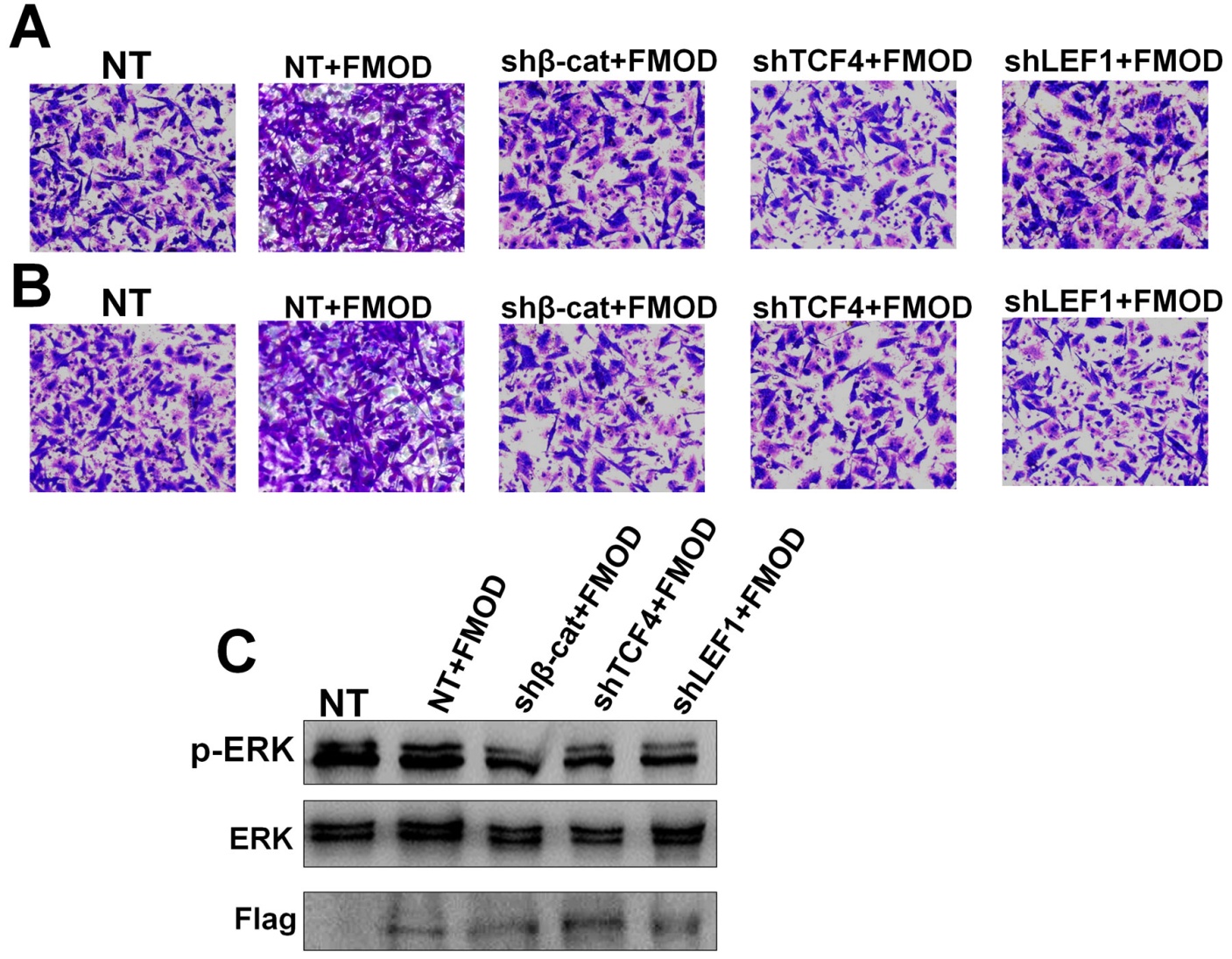


**Figure S2. β-catenin, TCF4, and LEF1 are required for BCCMI** **and ERK activation** **whereas overexpression of FMOD bypasses the requirements.** Overexpression of FMOD overrides the inhibitory effects on breast cancer MDA-MB-231 cell migration (A), invasion (B), and p-ERK (C) caused by β-catenin, TCF4, and LEF1 knock-down.
